# Perfect Absorption and Strong Coupling in Supported MoS_2_ Multilayers

**DOI:** 10.1021/acsnano.2c08947

**Published:** 2023-02-17

**Authors:** Adriana Canales, Oleg Kotov, Timur O. Shegai

**Affiliations:** Department of Physics, Chalmers University of Technology, 412 96 Göteborg, Sweden

**Keywords:** MoS_2_, TMD, perfect absorption, ultrathin films, strong coupling, exciton-polaritons, Fourier plane spectroscopy

## Abstract

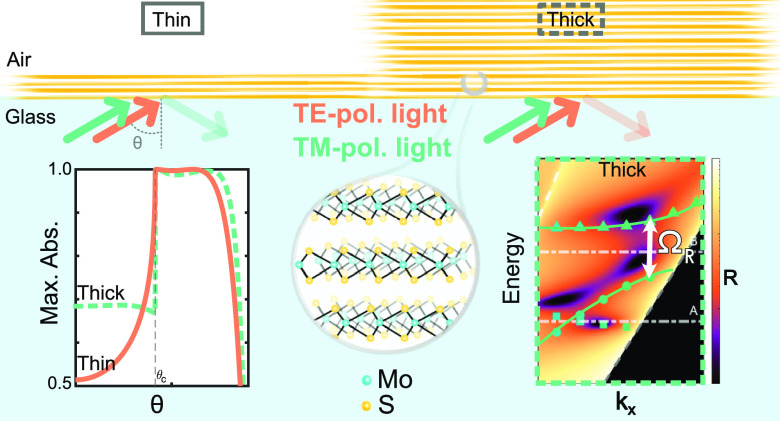

Perfect absorption and strong coupling are two highly sought-after regimes of light–matter interactions. Both regimes have been studied as separate phenomena in excitonic 2D materials, particularly in MoS_2_. However, the structures used to reach these regimes often require intricate nanofabrication. Here, we demonstrate the occurrence of perfect absorption and strong coupling in thin MoS_2_ multilayers supported by a glass substrate. We measure reflection spectra of mechanically exfoliated MoS_2_ flakes at various angles beyond the light-line via Fourier plane imaging and spectroscopy and find that absorption in MoS_2_ monolayers increases up to 74% at the C-exciton by illuminating at the critical angle. Perfect absorption is achieved for ultrathin MoS_2_ flakes (4–8 layers) with a notable angle and frequency sensitivity to the exact number of layers. By calculating zeros and poles of the scattering matrix in the complex frequency plane, we identify perfect absorption (zeros) and strong coupling (poles) conditions for thin (<10 layers) and thick (>10 layers) limits. Our findings reveal rich physics of light–matter interactions in bare MoS_2_ flakes, which could be useful for nanophotonic and light harvesting applications.

## Introduction

Maximization of light absorption while minimizing the thickness of the absorbing layer is one of the most important tasks of modern optics and a challenge for engineering implementation. The materials and structures, which exhibit perfect absorption in ultrathin films, are rather diverse.^1^ Generally, perfect absorption occurs when all radiation is captured by an absorbing film, which means complete suppression of both reflection and transmission. However, this cannot be achieved by simply increasing the loss function of the material itself, since it will entail an increase in the scattering of light. One way to overcome this is to use an asymmetrical surrounding with a suppressed transmission channel. The latter is usually implemented with a backside mirror (Salisbury screen) or a prism substrate enabling realization of the total internal reflection (TIR) scheme.^2−5^ Then, the perfect absorption corresponds to the complete suppression of the reflection, which is typically achieved by satisfying the critical-coupling condition (the radiative loss balances the nonradiative one). The critical-coupling condition can be fulfilled for homogeneous thin films with sufficient thickness and material losses only for some discrete frequencies and angles of incidence. At the same time, angular- and polarization-tolerant operation of thin absorbers even for normal light incidence can be realized using plasmonic nanostructures^6−9^ or impedance-matched metamaterials,^10^ which are quite complex solutions from a nanofabrication point of view.

In excitonic thin films, enhanced absorption has been reported for *J*-aggregates in the backside mirror configuration,^11^ even reaching perfect absorption.^12^ The latter has also been observed in the TIR configuration.^4,5^ The high oscillator strength and intrinsic losses of excitons in transition metal dichalcogenides (TMDs) enhance light–matter interactions.^13^ Therefore, several studies have focused on enhancing TMDs absorption using various fabrication strategies,^14−16^ reaching nearly perfect absorption even in monolayers.^17−19^ Importantly, the same excitonic characteristics make TMDs great candidates also for strong light–matter coupling.

In the strong coupling or polaritonic regime of light–matter interactions, the material excitation (in our case exciton) exchanges energy with the photonic mode faster than the individual subsystem decay rates.^20,21^ This generates light–matter hybrid states called exciton-polaritons with new eigenenergies and decay rates, which potentially may be used for nonlinear optics applications,^22^ as well as for modification of material properties.^23^ Following these ideas, exciton-polaritons in TMDs have been realized using external optical resonators, such as Fabry-Pérot (FP) microcavities and plasmonic nanoparticles.^24−31^ Recently, however, polaritons have been also realized by hybridizing excitons with photonic modes supported by the structure and three-dimensional geometry of the material itself.^32−36^ Such *cavity-free* exciton-polaritons have been experimentally observed in TMDs by coupling to self-sustained FP and Mie resonances,^37−40^ as well as to planar waveguide modes.^41−44^ However, observation of strong coupling and perfect absorption in the same structure has not been reported to date.

Here, we demonstrate that both strong coupling and perfect absorption can be found in the same MoS_2_ multilayer flake supported by a glass substrate. We use the scattering  matrix formalism^45^ to theoretically identify perfect absorption (zeros of -matrix) and polaritonic eigenmodes of the system (poles of -matrix). Our experimental approach does not require complex nanofabrication, as it is based on measuring reflection of MoS_2_ flakes on glass beyond the light-line (LL) by optical microscopy Fourier plane imaging and spectroscopy. Both phenomena, perfect absorption and strong coupling, in our study are ultimately enabled by the high oscillator strength of excitons in MoS_2_ and their intrinsic losses, and are thus anticipated to appear in other layered materials of the TMD family.

## Results

### Measuring MoS_2_ Optical Properties beyond the Light-Line

**Figure 1:**
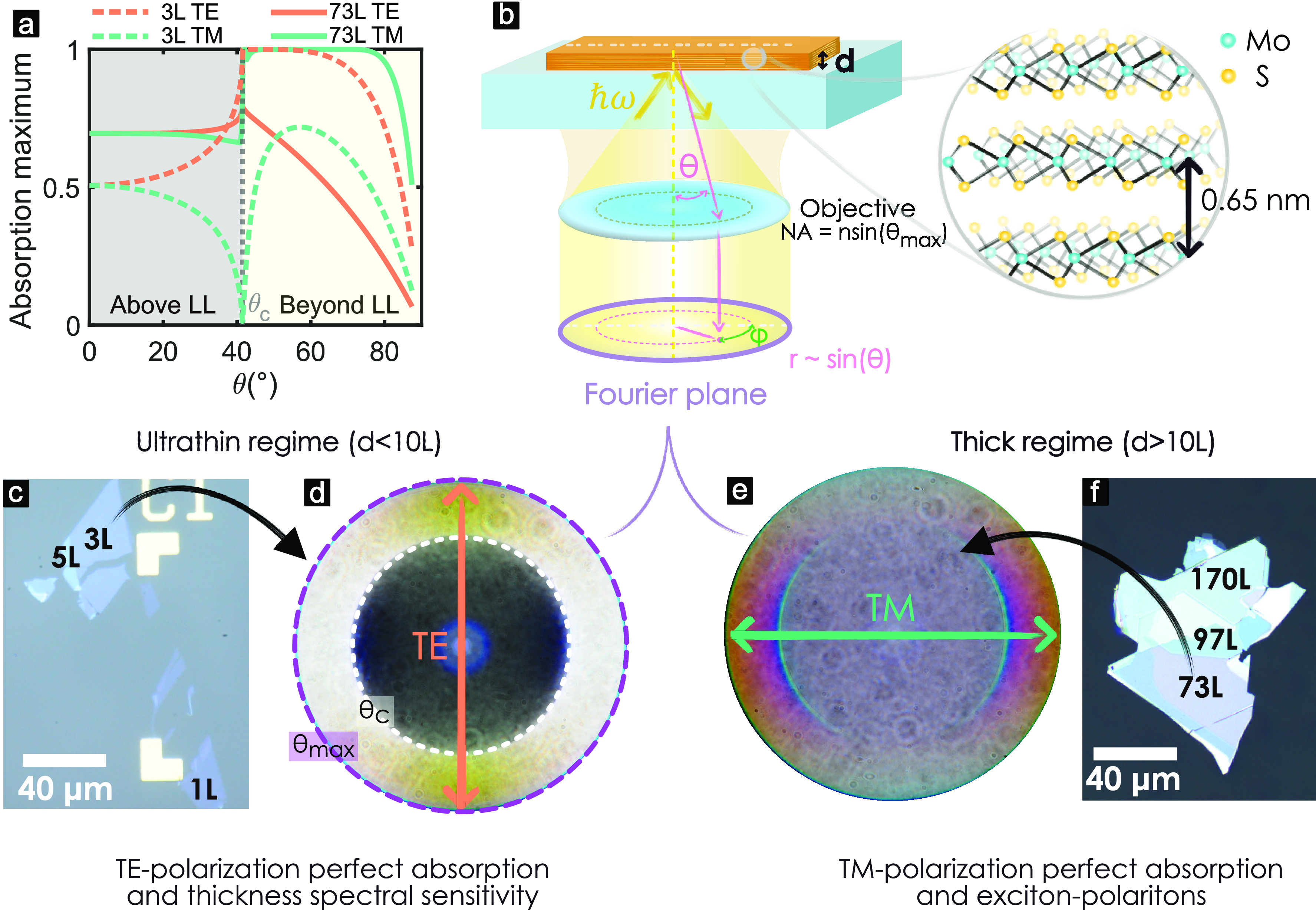
Optical measurements of MoS_2_ above and beyond the light-line (LL). (a) Calculation of absorption maxima for 3L and 73L in the visible range (ℏω ∈ [1.6, 2.9] eV) above and beyond the LL. TE absorption is higher than TM for 3L and, vice versa for 73L. (b) Scheme of experimental setup used to image the Fourier plane of the objective. Light is focused at high angles through an oil immersion objective (60×, NA = 1.49) on a MoS_2_ flake of thickness *d*. Through the same path, the Fourier plane (in purple) is imaged to measure angle-resolved reflection spectra for both TE and TM polarizations (see Figure S1). In the Fourier plane, the angular information is distributed along the radial direction as *r* ∝ sin θ. The structure of MoS_2_ is shown to the right, marking the interlayer distance. (c) Bright-field (BF) image in reflection of thin slabs of MoS_2_ transferred on glass, highlighting the number of layers. (d) True-color image in reflection of the Fourier plane of a thin slab (3L, *d* ∼ 2 nm). The dashed white circle marks the critical angle (θ_c_ = 41.5°). TE-polarized spectra are measured along the orange arrow. The maximum angle that can be measured is shown as a purple dashed line and is determined by the numerical aperture (NA) of the objective. (e) Fourier plane true-color image of a thick slab (73L, *d* ∼ 47.5 nm). TM-polarized spectra are measured along the green arrows. (f) BF image in reflection of several thick MoS_2_ flakes, including the 73L slab.

The essence of this study is summarized in Figure 1a, where the maximum value of the absorption in the visible (1.6–2.9 eV) was calculated for different angles for transverse electric (TE, in orange) and transverse magnetic (TM, in green) polarizations in two regimes: ultrathin (3L, *d* ∼ 2 nm) and thick flakes (73L, *d* ∼ 47.5 nm). The dashed lines represent the behavior of ultrathin slabs and the solid lines of the thick ones. Beyond the critical angle, absorption is maximized for TE polarization in 3L flakes, whereas for 73L flakes TM-polarized light is perfectly absorbed. This motivates our interest in measuring absorption at angles greater than the air/glass critical angle, θ > θ_c_ = 41.5°, in both TE and TM polarization channels.

When illuminating from the glass side, at angles beyond the critical angle, we satisfy the condition of TIR. Therefore, we can measure reflection and relate it to absorption as, *A* = 1 – *R*. Using an oil immersion objective allows one to measure reflection from micron-sized samples at θ > θ_c_ = 41.5° (see Methods). To measure MoS_2_ spectra with angle resolution, we used Fourier plane spectroscopy and microscopy,^46^ as shown in Figures 1b and S1. The Fourier plane imaging allows expanding the angular information such that the radial direction contains information about the polar angle dependence of the reflection, *r* ∝ sin θ (Figure 1b), while the tangential direction encodes polarization. Specifically, TE and TM polarizations can be distinguished by measuring along the arrows shown in Figure 1d,e (see Supporting Information (SI) section 1 for details).^46^

The difference between absorption in ultrathin and thick multilayers is clearly visible in the Fourier plane true-color images (Figure 1d,e). The Fourier plane of ultrathin MoS_2_ slabs (Figure 1d) is similar to the one of a bare glass substrate (Figure S1). In this case, light is mostly transmitted for θ < θ_c_, explaining the dark area in Figure 1d. Beyond θ_c_, TIR occurs, appearing as the bright area in Figure 1d. The difference between glass and ultrathin MoS_2_ can be seen in the TE polarization direction (orange arrow), where absorption enhancement appears as a shadow in the TIR region.

On the other hand, thick MoS_2_ slabs reflect light in accordance with FP modes sustained by these structures, which results in vivid colors (Figure 1f). These colors and their angular dispersion can be visualized in the Fourier plane (Figure 1e), particularly along the TM polarization direction (green arrow). The information contained in the Fourier plane, including perfect absorption and strong coupling will be discussed in following sections.

### Absorption Enhancement in a Monolayer

**Figure 2:**
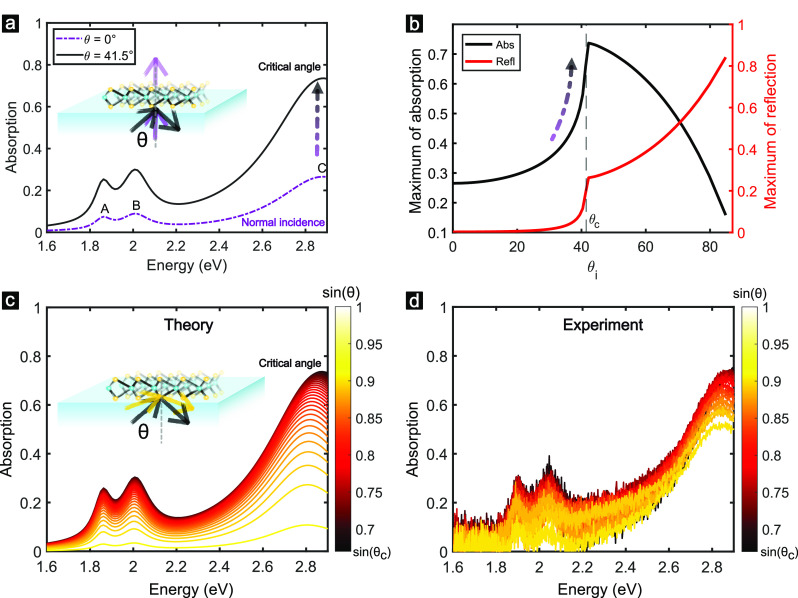
Monolayer absorption enhancement of TE-polarized light. (a) Absorption calculation of a MoS_2_ monolayer at normal incidence (dashed-dotted line) and at the critical angle θ_c_ = 41.5° (solid line). The absorption given by A-, B-, and C-excitons is marked. (b) Calculated maximum of absorption and maximum of reflectivity in the visible (ℏω ∈ [1.6, 2.9] eV) depending on the angle of incidence. (c) Calculated absorption spectra for angles beyond the LL, θ ∈ [θ_c_, 90°]. (d) Experimental absorption beyond the LL, θ ∈ [θ_c_, 74°]. The colormap shows the angle, sin(θ), of each spectrum.

At normal incidence a MoS_2_ monolayer on a glass substrate can absorb ∼29% of light around the C-exciton band.^47^ This is illustrated in Figure 2a, which shows calculated absorption spectra with a dotted line. Intricate structures have been used previously to enhance this absorption to nearly perfect absorption.^15,16,19,48^ In our study, the absorption of TE-polarized light is enhanced by simply increasing the angle of incidence to the critical angle, θ_c_, (Figure 2b) as predicted previously.^3^ The increase in absorption depends on the energy of the photon and the angle of incidence (Figure 2c). At θ_c_ the absorption increases on average ∼3.4× for all photon energies. Around the C-exciton band the absorption reaches 74% (dotted arrow in Figure 2a), providing substantial enhancement without any additional fabrication efforts.

Beyond the LL, θ > θ_c_, the reflection increases, while the maximum of absorption decreases with the angle (Figure 2b,c). The angular spectral response was calculated for TE-polarized light (Figure 2c) and shows that the maximum in absorption occurs always at the C-exciton. This was verified experimentally by measuring spectra for various angles (sin θ) for TE polarization, Figure 2d. Our experimental results are in good agreement with theoretical calculations. For TM polarization the maximum absorption is achieved at θ = 55° and reaches only 35% at the C-exciton (Figure S6), while the reflection reaches on average 99.9% in the visible range (1.6–2.9 eV) at θ_c_ (Figure S6a).

### Perfect Absorption in Thin Flakes

**Figure 3:**
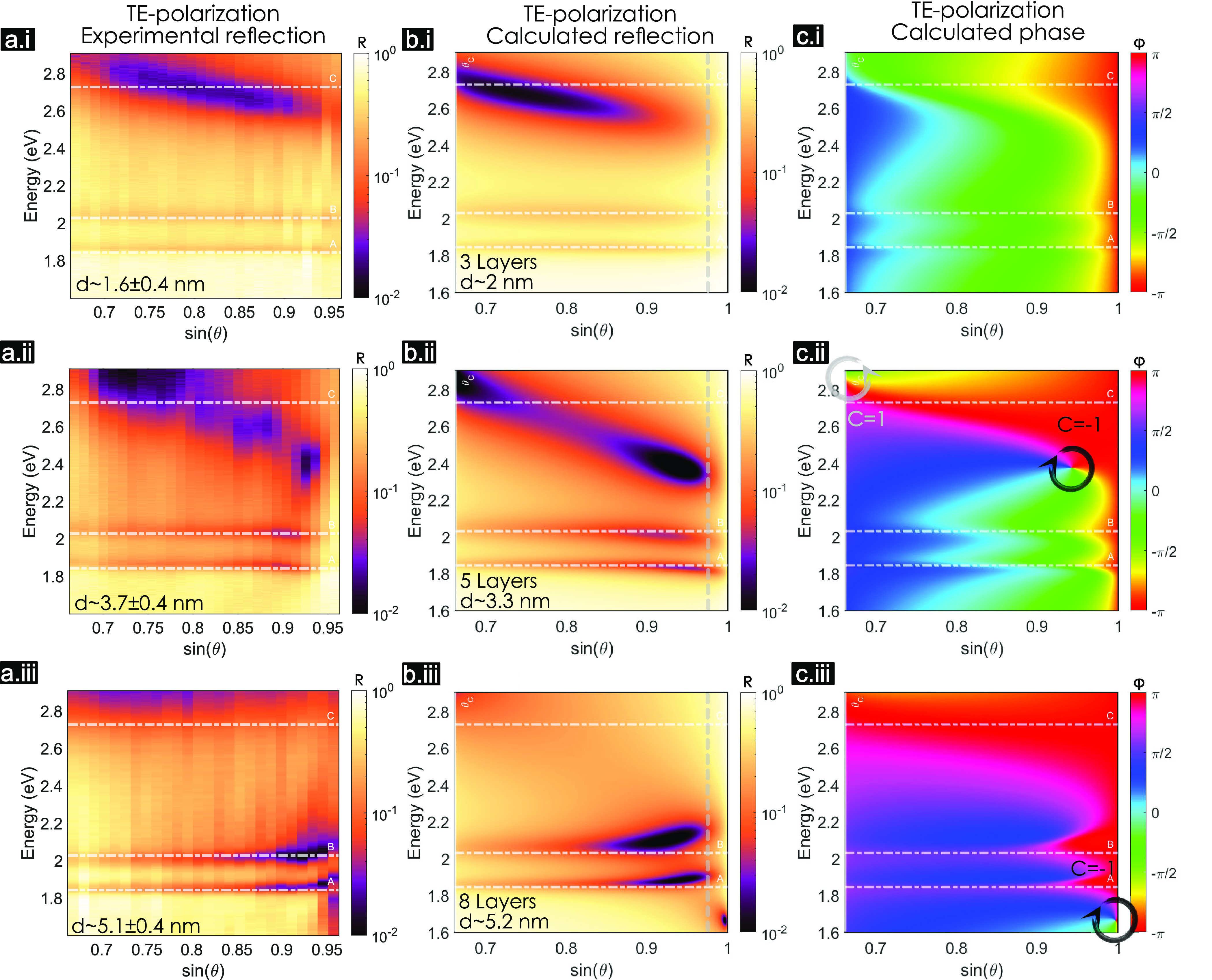
Thickness-dependent perfect absorption in thin MoS_2_ slabs. Reflection spectra at various angles beyond the LL: (a) measured experimentally (the thickness, *d*, measured by atomic force microscopy (AFM; see Figure S2) is marked in each plot) and (b) calculated using the transfer matrix method (TMM). (c) Calculated phase of the reflected wave, ϕ, shows singularities in the points of perfect absorption. Gray and black circles mark the topological charge of the singularities (1 and −1, respectively). Note the *x*-axis difference between experiment and theory, the gray dashed line in (b) marks the limit of the experiment in (a). The sensitivity of dispersion to thickness is shown for slabs of (i) 3L, (ii) 5L, and (iii) 8L.

Similar to the monolayer, absorption of TE-polarized light is enhanced up to 90% in bilayers (Figure S7b) and 99% in trilayers (Figure 3i). For 4–8 layers (2.6–5.2 nm), the slabs show perfect absorption in the visible (Figure 3ii,iii). There is good agreement between experiment (Figure 3a) and calculation (Figure 3b), considering that the experimental resolution is ∼1% (see Methods). Due to the experimental resolution limit, we additionally distinguish the 99% absorption from true perfect absorption by calculating the phase of the reflected light wave in Figure 3c.

The phase of the reflected wave, ϕ, where *E*_ref_/*E*_inc_ = |*r*|*e*^*i*ϕ^, is not defined when the amplitude of the reflected field is zero, giving rise to a singularity point.^9,45,49^ These singularities, illustrated in Figure 3c, correspond to the frequencies of the -matrix zeros crossing the real frequency axis^9^ (see Methods). Singularities come in pairs for thin slabs, as shown in Figure 3c,ii and SI section 3.3. There is one pair per excitonic resonance (Figure S9), and they have opposite topological charges, as observed previously in metasurfaces.^9^ The behavior of the pairs of singularities in thin flakes is further illustrated via the zeros of the -matrix in Figure S9 and SI section 3.3. The topological protection of such singularities guarantees that they are robust to small imperfections or variations in the structure.^50^

To analytically determine the frequency and angles (ω, sin θ) of perfect absorption depending on the thickness of the slab, *d*, we consider a three-layer system where the incident medium has a higher permittivity than the outgoing one, ε_1_ > ε_3_ and θ > θ_c_. Perfect absorption occurs in the middle layer with complex permittivity  when . Using the Fresnel coefficients *r*_12,23_ for TE-polarized light and approximating for thin flakes, *k*_2_*d* ≪ 1 and ε_1,3_ ≪ ε (see SI section 3.4), we obtain that perfect absorption occurs when
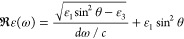
1
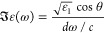
2

Equations 1 and 2 are satisfied simultaneously in the points of perfect absorption. These relations can be used as *universal scaling laws* of perfect absorption in thin films in TIR configuration. Their full derivation can be found in section 3.4 of the SI.

In this case, ε_1_ = 1.51^2^, ε_3_ = 1, and ε is given by eq 4 (see Methods). Figure S10b shows the five intersections of both equations for 7L, which correspond to the five phase singularity points (Figure S10a) and the zeros of the -matrix in Figure S9. Combining eqs 1 and 2, as in Figure S10d, allows one to find all possible angles and frequencies at which the perfect absorption condition is satisfied for various numbers of layers, *N*. There it is shown that 8L is the thickest slab to perfectly absorb TE-polarized light in the visible spectral range. Furthermore, 4L is the thinnest slab that reaches perfect absorption in MoS_2_. A hypothetical material with higher loss and oscillator strength could present perfect absorption in thinner slabs, even down to a monolayer limit. However, MoS_2_ has the highest oscillator strength and intrinsic loss (around the C-exciton) among conventional TMDs.^47,51^ Therefore, we expect perfect absorption in other TMDs (of general formula MX_2_, where M = Mo, W and X = S, Se, Te) to occur only in thicker slabs.

The frequency and angle at which perfect absorption occurs has high sensitivity to thickness for thin slabs (*d* < 9L). Thus, the dispersion of reflection varies substantially with the number of layers, as shown in Figure 3i–iii. In fact, the angular spectrum changes so much that it is possible to optically determine the thickness with a single-layer precision. Figure S7 shows calculations of angular spectra for every number of layers in the 1L–9L range and Figure S8 its experimental counterpart. This sensitivity occurs in a broader thickness range than accessible by Raman spectroscopy, which is routinely used to distinguish the number of layers below 6L.^52^ Moreover, given the uniqueness of each angular spectrum there is no need to compare the results with nearby monolayers to calibrate the thickness of a given flake, as has to be done using techniques such as Raman spectroscopy, AFM, or optical contrast.^53^ The dispersion spectra thus provide an unambiguous optical spectroscopy method to directly identify the number of layers in thin MoS_2_ flakes without the necessity of comparing to neighboring flakes.

### Perfect Absorption and Strong Coupling in Thick Flakes

Increasing the number of layers beyond 8L results in TE polarization no longer being perfectly absorbed. However, in this section we show that above 10L TM-polarized light is perfectly absorbed for all thicknesses. In this case, the angles and frequencies at which prefect absorption occurs depend on the interaction between excitonic and photonic modes in the flakes.

**Figure 4:**
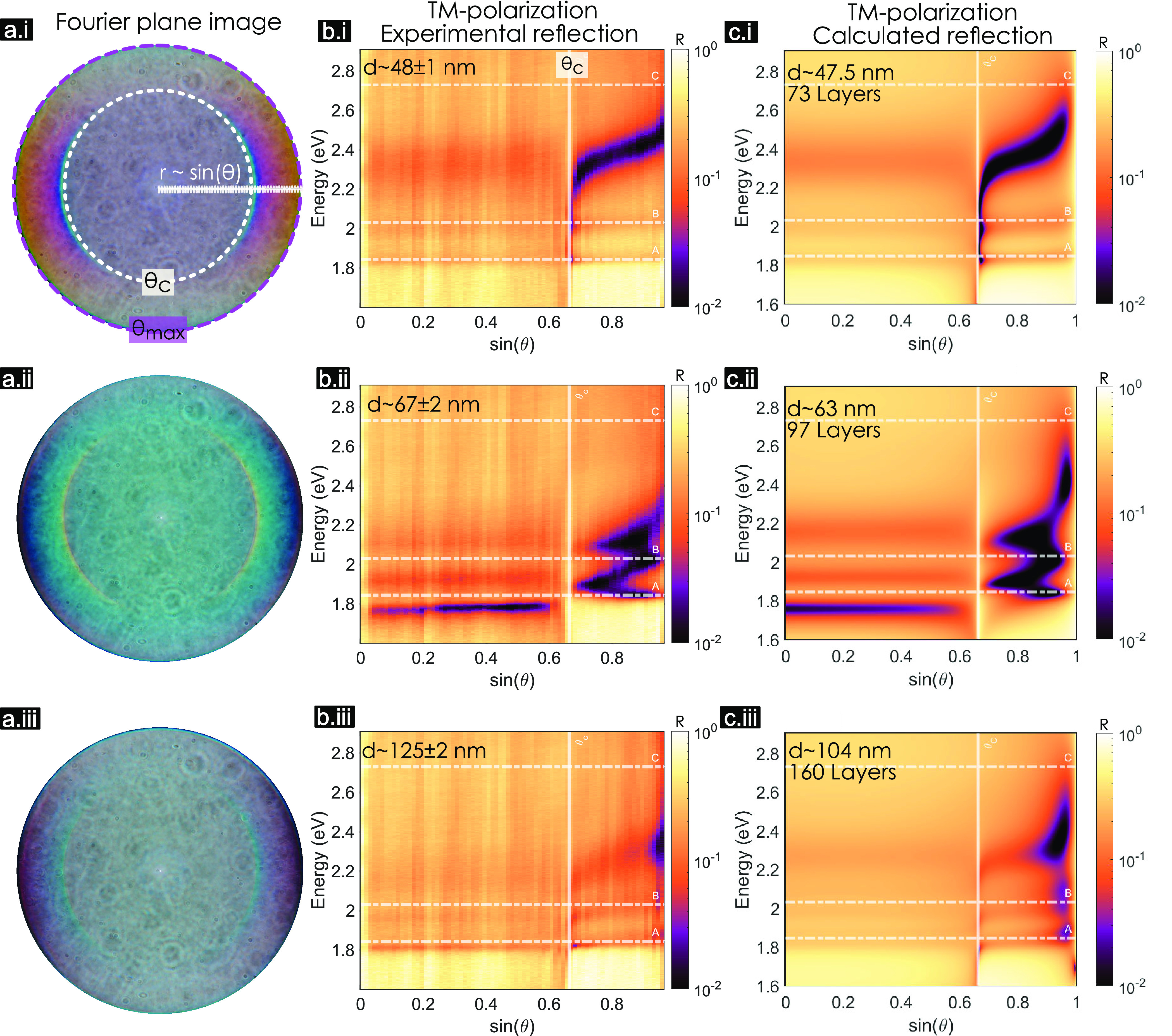
Perfect absorption in thick MoS_2_ slabs. (a) True-color image of the Fourier plane in reflection of thick flakes of increasing thickness (i–iii). The dotted white and purple circles mark the critical angle, sin θ_c_, and maximum measurable angle. The white line marks the region along which the spectra were taken. (b) Measured reflection spectra of TM-polarized light. The thickness, *d*, measured by AFM (see Figure S4) is marked in each plot. The vertical white line marks sin(θ_c_). The dashed lines indicate the frequency of the excitons. (c) Calculation of reflection spectra for TM polarization for angles up to 90° considering isotropic MoS_2_. The thicknesses are (i) experimental: 48 ± 1 nm, calculation: 73L (47.5 nm); (ii) experimental: 67 ± 2 nm, calculation: 97L (63 nm); (iii) experimental: 125 ± 2 nm, calculation: 160L (104 nm). The differences between calculations and experiments decrease when considering the effect of dielectric function anisotropy.

The high refractive index of MoS_2_ allows thick flakes to sustain FP resonances. These resonances result in the colorful appearance of thick MoS_2_ flakes shown in BF images in Figure 1f. The same resonances appear in the Fourier plane (Figure 4a). The color close to the center is a result of the FP mode above the LL. The rainbow region visible beyond the critical angle (white dashed line) is caused by the interplay between the excitons and the photonic mode, which in some cases results in exciton-polaritons.

To understand the nature of these modes, we measured the angular reflectivity spectra of several thicknesses, as shown in Figure 4b. Here, the FP mode appears as a broad minimum in reflection above the LL (sin θ < sin θ_c_); see Figure 4b,i. Beyond the critical angle, the photonic mode is narrower and deeper, even reaching perfect absorption. Therefore, for all thicknesses in Figure 4, phase singularities are present for θ > θ_c_ (Figure S11). In general, all thicknesses above 10L show at least one point of perfect absorption in the studied energy range for TM polarization. Unfortunately, it is hard to demonstrate this analytically because the condition *k*_2_*d* ≪ 1 is not satisfied for thick flakes, which support perfect absorption of TM-polarized light. However, we demonstrate this by numerical calculations in Figure S12. Additionally, the interplay between the photonic and excitonic resonances determines the angle and frequency of perfect absorption for each flake thickness (Figure S12).

Perfect absorption can be observed for both TM and TE polarizations in the same flake in a small window of thicknesses between 110L and 125L (71–82 nm), when the FP mode is close to the A-exciton. However, the exact angles and photon energies at which the perfect absorption conditions are satisfied are polarization dependent (see Figure S13).

Note that the thickness of the experimental (Figure 4b) and calculated angular spectra (Figure 4c) are somewhat different, especially for the thickest studied flake. In calculations, the thicknesses of the flakes were chosen such that the calculated angular spectra closely resembled the measured ones. We expected some differences because the MoS_2_ permittivity in these calculations was assumed isotropic. However, MoS_2_ is an anisotropic van der Waals material^51^ and it may be important to take this into consideration for TM polarization. Thus, the effect of anisotropy was systematically accounted for in Figure S13. Interestingly, however, the angular spectra for the same flake thickness vary only modestly when comparing isotropic and anisotropic permittivities. We note that the out-of-plane refractive index of MoS_2_ is *n*_⊥_∼ 2.74 and is weakly dispersive, causing a birefringence of around Δ*n* = 1.34 in the near-infrared spectral range.^51^ The modest effect of anisotropy in this case can be assigned to the high values of *n*_∥_, which cause substantial refraction in the material and in doing so diminish the effect of birefringence. Working with the isotropic dielectric function, on the other hand, has an advantage of allowing to derive simple analytical formulas for TE polarization (SI section 4.4) and a simpler approach to the complex frequency plane problem (as we show below).

To observe the Rabi splitting as a result of strong light–matter coupling, the photonic and excitonic modes should have similar frequencies, ω_*c*_ ∼ ω_0,*i*_. The frequencies of A- and B-excitons are marked by dash-dotted white lines in Figure 4b,i. Therefore, the photonic modes (reflectivity minima) should be red-shifted by increasing the thickness of the slab. Figure 4ii shows the Fourier plane and dispersion of a flake with almost zero photon-exciton detuning. Increasing the thickness further results in higher-order FP modes interacting with excitons as in Figure 4iii.

From Figure 4b,ii one could naively conclude that both A- and B-excitons are strongly coupled with the FP mode above the LL (θ < θ_c_) because the splitting in reflection is visible. Nevertheless, to ensure strong coupling, it is important to observe the splitting in absorption^38,54,55^ or in the eigenenergies of the structure.^32^ Therefore, the next section is focused on the eigenmode analysis of a 97L slab (Figure 4ii) in the complex frequency plane, showing that B- and C-excitons are indeed strongly coupled to the photonic mode both above and below the LL. That section also shows that in addition to strong coupling, this particular 97L MoS_2_ slab, just like other thick slabs, supports perfect absorption of TM-polarized light.

### Perfect Absorption and Strong Coupling in the Complex Frequency Plane

**Figure 5:**
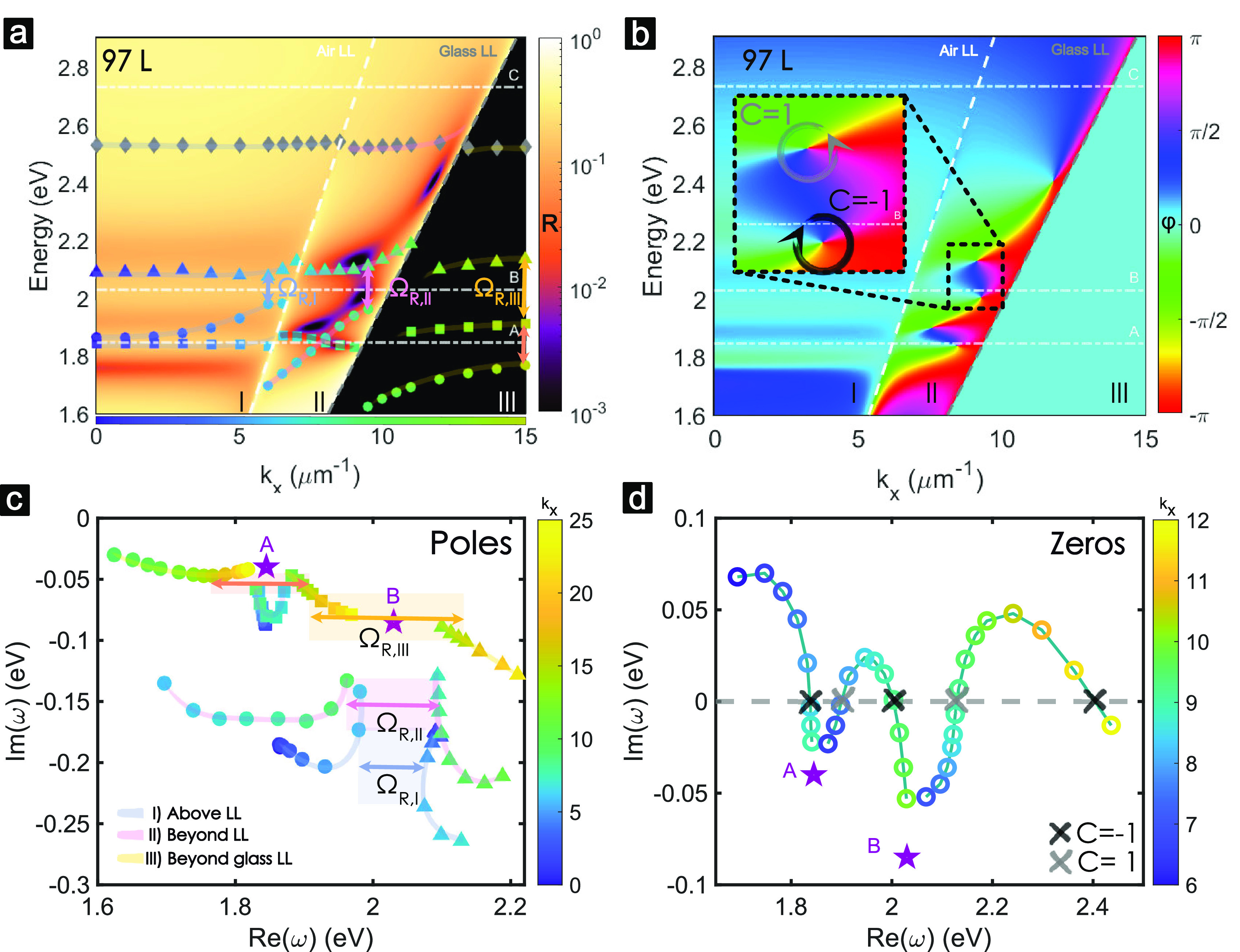
MoS_2_ strong coupling (poles) and perfect absorption (zeros) in the complex frequency plane for TM polarization. (a) Calculated reflection dispersion (ω vs *k*_*x*_ = *nk*_0_ sin θ) of a 97L MoS_2_ layer (experimental counterpart is shown in Figure 4ii) with the QNMs eigenfrequencies (poles of -matrix) plotted with markers on top. The markers represent different families of modes, which can be excitonic, photonic, or polaritonic. The Rabi splitting in each region is marked in arrows for A- (orange) and B-excitons (blue, pink, and yellow). (b) Phase of the reflected wave showing the singularity points in between both LLs. The inset shows the singularities close to the B-exciton. The gray circle marks the one with topological charge *C* = +1 and the black circle with *C* = −1. (c) Trajectories of the poles in the complex−ω plane for various wave vectors, *k*_*x*_, in different colors help to visualize the losses. The trajectories corresponding to different regions are connected with different colors (blue for I, pink for II, and yellow for III). The Rabi splitting is marked once more with arrows of the corresponding colors and with Ω_R_, the shadow square marks the points with *k*_*x*_ where the Rabi splitting happens. (d) Zeros’ trajectories in the complex−ω plane for different *k*_*x*_. The real axis (dotted gray line) is crossed five times, corresponding to the singularity points in (b). The points with topological charge *C* = +1 and *C* = −1 are, respectively, marked with gray and black crosses.

Figure 5a shows a calculation of dispersion in reflection of a 97L MoS_2_ slab that corresponds to the experiment shown in Figure 4b,ii. This is the same data as in Figure 4c,ii, but with the reflection resolution increased to 10^–3^ and in a representation that also shows the momentum space beyond the glass LL. There are three distinct regions: (I) above the air LL (white dashed line), (II) below the air LL but above the glass LL (gray dashed line), and (III) below the glass LL. Fourier plane microscopy allows one to measure reflectivity in regions I and II, as we do here. Region III can be probed by (scattering-type scanning near-field optical microscopy) *s*-SNOM.^41,42^

To shed light on strong coupling and perfect absorption occurring in the same MoS_2_ flake, we analyzed the zeros and poles of the system’s scattering matrix in the complex frequency plane^45,56^ (see Methods). Perfect absorption happens when the -matrix zeros cross the real-axis. The angles and frequencies corresponding to this crossing are then determined through *k*-vector and ω dependence of the -matrix. Similarly, the -matrix poles correspond to the complex eigenfrequencies of the quasinormal modes (QNMs) of the structure.^45,57^

Symbols in Figure 5a depict the calculated QNMs of the system. The color variation from blue to yellow corresponds to increasing *k*_*x*_ = *nk*_0_ sin θ. The QNMs are photonic, excitonic, or polaritonic depending on the region and the light–matter coupling regime. Polaritons lift the degeneracy of the photonic and excitonic modes at zero detuning (ω_c_ = ω_0_). Then the new eigenfrequencies, ω_±_, are separated by the Rabi splitting (in this text, we refer to Rabi splitting as the difference of the polaritonic eigenfrequencies at zero detuning only), which according to the coupled oscillators model reads
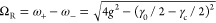
3Here, *g* is the coupling strength, while γ_0_ and γ_c_ are intrinsic excitonic and cavity losses, respectively. Rabi splitting has real values upon passing the exceptional point at *g* > |γ_0_ – γ_*c*_|/4. Furthermore, the polaritonic eigenstates may be spectrally resolved when the Rabi splitting exceeds the polaritons’ losses, Ω_R_ > γ_±_ = (γ_c_ + γ_0_)/2.^20^

The arrows in Figure 5a mark the different Rabi splittings due to interactions between various photonic modes and the B-exciton. In region I, Ω_R,I_ = 0.096 eV (blue arrow). In region II, Ω_R,II_ = 0.15 eV (pink arrow). In region III, there is strong coupling with both A- and B-excitons, and the Rabi splitting with the B-exciton is Ω_R,III_ = 0.22 eV (yellow arrow). The mismatch in the observed Rabi splittings, Ω_R,I_ < Ω_R,II_ < Ω_R,III_, is partly due to the loss mismatch between excitonic and photonic components and partly due to difference in the coupling strength in different regions. Losses can be visualized in the complex frequency plane by the imaginary part of the QNMs,  (Figure 5c). For example, the uncoupled excitons (purple stars in Figure 5c) are positioned in the imaginary axis in accordance with their intrinsic losses, γ_0,*i*_/2 with *i* = {*A*, *B*}, while polaritonic line widths are given by . Of course, the Rabi splitting also depends on the coupling strength, *g* (which in turn depends on the exciton oscillator strength, exciton confinement and mode volume), which varies in each region and cannot be directly calculated using the QNMs approach. However, it can be estimated using a simplified model following eq 3—the Rabi splitting is obtained from the QNMs, the excitonic loss γ_0,B_ = 0.17 eV is obtained from the Lorentz fit (Table 1), and to estimate the photonic losses γ_c_, we use  (marked arrows in Figure 5c). Then we estimate intrinsic photonic losses as γ_c_ = 2γ_±_ – γ_0_, such that γ_c,I_ ∼ 0.63 eV, γ_c,II_ ∼ 0.45 eV, and γ_c,III_ ∼ 0.14 eV. As a result, the coupling strengths in the three regions are *g*_I_ ∼ 0.13 eV, *g*_II_ ∼ 0.1 eV, and *g*_III_ ∼ 0.11 eV, which are all below the bulk coupling strength limit^32^ for B-excitons in MoS_2_,  eV (Table 1).

**Table 1:** Values for the Lorentzian Fit of the Bulk Permittivity of MoS_2_^47^ (See Figure S5 for the Fitting) and Calculated Bulk Coupling Strength (32)

exciton	ε_*∞*_	*f*ω_P_^2^ (eV^2^)	ω_0_ (eV)	γ_0_ (eV)	*g*_B_ (eV)
A-bulk	13	1.53	1.845	0.08	0.17
B-bulk	13	3.38	2.03	0.17	0.25
C-bulk	13	47.92	2.73	0.55	0.96
A-mono	10	1.56	1.86	0.09	0.2
B-mono	10	3.64	2.01	0.15	0.3
C-mono	10	50.59	2.88	0.46	1.12

The differences between various light–matter interaction regimes in regions I and II can be visualized in the complex frequency plane.^32^ As shown in Figure 5c, the weakly coupled A-exciton-like modes (squares) move around the uncoupled A-exciton mode (purple star), being only slightly perturbed by the presence of the photonic mode(s) (circles). In the case of B-excitons, the behavior is drastically different—the larger oscillator strength of the B-exciton (Table 1) increases the coupling strength and the trajectories split in two: the lower polariton (circles) and the upper polariton (triangles) giving rise to a Rabi splitting. This is observed as anticrossing in dispersion (Figure 5a), where the Rabi splittings are marked with arrows of the same colors as in Figure 5c. In region III, the split trajectories of the QNMs show that both A- and B-excitons are strongly coupled. However, Ω_R,A_ = 0.14 eV  since the oscillator strength of the A-exciton is smaller. Furthermore, in the spectral range of Figure 5a, only the lower edge of the coupled C-exciton is visible and marked with diamonds, but the high loss of the C-exciton together with its high oscillator strength (Table 1) substantially increase the loss of the B-exciton-polaritons. In fact, based on the calculated values of the C-exciton bulk coupling strength, we anticipate it to be in the ultrastrong coupling regime (Table 1).

On the perfect absorption side, Figure 5b shows the phase singularities that appear when reaching perfect absorption in region II. These points correspond to the -matrix zeros crossing the real axis (gray dashed line) in Figure 5d. The zeros related to photonic-like modes (low loss) usually appear in the upper-half of the complex frequency plane. The zeros related to excitonic modes appear in the lower-half due to their high losses. In this case, the trajectories of the zeros cross the real frequency axis several times because of the light–matter interactions. Note that singularities arise for both weak and strong coupling, as shown in Figure 5b,d, and that perfect absorption points do not coincide with polaritonic eigenmodes (see SI section 4.6).

It should be noted that all the analysis in Figure 5 was performed assuming isotropic dielectric function of MoS_2_ fitted by a Lorentz model (see methods). However, Figure S15a shows that if the anisotropy is taken into account, the poles still behave very similarly to Figure 5 and that the Rabi splitting is slightly larger in the anisotropic case. The topologically protected phase singularities are robust to the anisotropy, but they appear at slightly different energies and angles (Figure S15b). Therefore, both strong coupling and perfect absorption are still observed and only modestly modified when anisotropy is taken into account. Summarizing, Figure 5 shows perfect absorption and strong light–matter coupling naturally coexisting in a simple MoS_2_ slab.

## Conclusion

TMDs, in particular, MoS_2_, have optical properties that make them interesting for perfect absorption and strong coupling regimes of light–matter interactions. Here, we showed that both effects can occur in the same bare MoS_2_ slab supported by a glass substrate. The observation of both phenomena does not require any fabrication beyond flake exfoliation and transfer. In this simple setup, even monolayers show enhanced absorption close to the critical angle, where the absorption can reach up to 74% at the C-exciton for TE polarization. Moreover, for TE-polarized light absorption increases to 90 and 99% for 2L and 3L, respectively. Additionally, we derived an analytical expression to find the frequencies and angles at which perfect absorption occurs for any material with a complex permittivity, . This expression explains the small window (4L–8L) of perfect absorption in thin MoS_2_ for TE-polarized light. Furthermore, the perfect absorption condition results in a notable sensitivity of angular-dependent reflection spectra on the number of MoS_2_ layers. Thus, measuring beyond the light-line can be used as an absolute optical thickness characterization technique in a few-layer regime, even when other methods lack sensitivity.

Flakes with more than 10L showed perfect absorption for TM-polarized light. The angle and frequency at which perfect absorption occurs vary because of the interplay between photonic and excitonic resonances. That interplay can result in strong coupling and perfect absorption in the same bare MoS_2_ flake. Using the -matrix poles, we showed the presence of cavity-free exciton-polaritons by calculating the eigenmode splitting. Tracing the zeros of the -matrix provides information about the topological charge of the singularities given by the perfect absorption. To our knowledge, we presented the simplest structure that supports both strong coupling and perfect absorption regimes of light–matter interactions.

## Methods

### Calculations

Reflection (*R*) and transmission (*T*) spectra were calculated by the transfer matrix method (TMM)^58^ in a glass/MoS_2_/air structure, with illumination through the glass. The absorption was then calculated as *A* = 1 – *R* – *T*, where beyond the critical angle *T* = 0. The refractive index of glass and air were considered to be 1.51 and 1, respectively. The experimental permittivity of MoS_2_^47^ was fitted as a Lorentz material with 3 resonances as shown in eq 4 for a monolayer and for bulk. The values for the fitting are listed in Table 1.
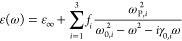
4

The quasi-normal modes (QNMs) are stationary solutions to the homogeneous Maxwell’s equations of an open system.^57^ In the text, *QNMs* and *modes* are used indistinctly although strictly speaking *modes* refer to solutions of closed systems. The QNMs of the system are found as poles of the eigenvalues of the -matrix in the complex−ω plane.^45^ Therefore, both frequencies of poles and zeros of the eigenvalues are complex. The real part of the eigenfrequencies of the poles is related with the frequency of the QNM and the imaginary part is related to its lifetime.^32^ Perfect absorption occurs when the frequencies of the zeros of the -matrix occur exactly at the real frequency axis, meaning that their imaginary part is zero.^56^ Both poles and zeros were calculated such that^59^
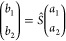
5where *a*_1,2_ and *b*_1,2_ denote the input and output wave amplitudes in both channels (1 for glass and 2 for air). The -matrix is then
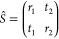
6Reflection and transmission coefficients on both channels, *r*_1,2_ and *t*_1,2_, were calculated using the TMM method.^58^ The reflection coefficient, |*r*_1_|^2^, and argument of *r*_1_ are plotted in Figures 2–5 to show perfect absorption. Then the eigenvalues of the -matrix are numerically computed and their poles and zeros are found by plotting their values in the complex frequency plane.

For simplicity we considered the permittivity of MoS_2_ to be isotropic. This is only reasonable because of the high in-plane refractive index of MoS_2_,^51^ refraction in the material is so high that even for TM-polarized light the electric field variation with angle is minimal. To take anisotropy into account one needs to make a standard substitution, such that  and ε_2_ → ε_∥_, where *k*_2_ is the wavevector in MoS_2_, ε_2_ is the isotropic permittivity given by eq 4, and ε_∥,⊥_ are the in-plane and out-of-plane components of the anisotropic permittivity. The small variations given by anisotropy are discussed in SI section S5.4.

### Sample Preparation

The samples were prepared on thin microscope glass (170 μm) coverslips (Menzel-Gläser #1.5 D 263 M). The coverslips were cleaned in acetone and isopropyl alcohol at 50 °C in ultrasonicator and then dried with compressed nitrogen, followed by O_2_ plasma cleaning.

The MoS_2_ flakes were mechanically exfoliated from a crystal (HQ Graphene) into a polydimethylsiloxane (PDMS) stamp. Then the flakes were transferred to glass coverslips via dry-transfer technique.^60^ Thicknesses of the studied flakes were measured in ambient conditions using a Bruker Dimension 3100 AFM in noncontact mode. The thickness was obtained with the Gwyddion software. The error bar is given by statistical analysis of several areas of the scan (see SI section 2).

### Optical Measurements

Dispersion measurements in reflection were performed using an inverted microscope (Nikon Eclipse TE2000-E) equipped with an oil immersion objective Nikon 60× and NA = 1.49 (Nikon CFI Apo TIRF 60XC oil, MRD01691). MoS_2_ flakes were illuminated with a laser-driven white light source (LDLS, EQ-99FC, high-brightness, flat-broadband spectrum) through the glass substrate. The oil immersion (Nikon NF 50 cm^3^, *n*_oil_ = 1.515) allows one to collect reflected light of angles up to sin θ_max_ = NA/*n*_*oil*_. The high magnification allows one to measure micron-sized structures. The Fourier plane of the objective was imaged using a Bertrand lens and its images were recorded using a digital color camera (Nikon D300S). A scheme of the experimental setup is shown in Figure S1 with a more detailed description.

The spectra for different radii in the Fourier plane were collected simultaneously for several angles with a fiber bundle consisting of 19 fibers with 100 μm core (Andor SR-OPT-8002). The fiber bundle was coupled to a spectrometer (Andor Shamrock SR-500i, equipped with a CCD detector Andor Newton 920). The angular resolution for sin θ was 0.012.

The minimum reflection our setup can resolve is ∼1%. This was obtained by measuring the reflection of a glass substrate at the Brewster angle for TM polarization, where ideally *R* = 0 for all wavelengths. The spectroscopic reference (of white light) was taken for every angle on glass beyond the critical angle, where *R* = 1 for all wavelengths due to total internal reflection.

## Supporting Information Available

Experimental setup and Fourier plane details; MoS_2_ thickness characterization by AFM and optical contrast; permittivity of MoS_2_ for calculations; absorption of TM polarized light in a monolayer; angular spectral sensitivity in thin layers; zeros of -matrix in thin layers; full derivation of perfect absorption condition for thin films; phase singularities in thick layers of TM-polarized light; reflection of TE-polarized light by thick layers; perfect absorption of both TE- and TM-polarized light in the same MoS_2_ slab; anisotropic MoS_2_ response used for calculating of TM-polarized light reflection from thick multilayers; poles and phase singularities taking the anisotropic MoS_2_ permittivity into account; poles of TE polarization thick slabs and discussion of zeros in TM polarization response of thick layers (PDF)
