# Supplementary material for: Perfect Absorption and Strong Coupling in Supported MoS2 Multilayers
Source: ACS Nano. 2023 Feb 17;17(4):3401–11. doi: 10.1021/acsnano.2c08947 (PMC9979649; doi:10.1021/acsnano.2c08947)
Supplement: Supplementary file 1 — nn2c08947_si_001.pdf [file nn2c08947_si_001.pdf]

Supporting Information for "Perfect absorption and strong coupling in supported MoS_2 multilayers"

Adriana Canales,[1] Oleg Kotov,[1], and Timur O. Shegai[1*]

[1]Department of Physics, Chalmers University of Technology, 412 96, Göteborg, Sweden,
*Corresponding author; E-mail: timurs@chalmers.se.

Contents

1 Fourier plane microscopy and spectroscopy 2

2 MoS_2 Thickness characterization 3

3 Reflection and absorption calculations 4
3.1 Permittivity for calculations 4
3.2 Monolayer TM absorption 6

4 Angular spectral sensitivity in thin layers of MoS_2 7
4.1 Calculation of angular reflection spectra of TE-polarized light 7
4.2 Experimental angular reflection spectra of TE-polarized light 7
4.3 $\hat{S}$-matrix zeros in thin MoS_2 layers 8
4.4 Direct calculation of perfect absorption condition for thin films 10

5 Reflection of thick slabs (d>10L) 13
5.1 Phase singularities in thick layers of TM-polarized light 13
5.2 Reflection of TE-polarized light by thick layers 14
5.3 Perfect absorption of both TE and TM-polarized light in the same MoS_2 slab 16
5.4 Anisotropic MoS_2 calculation of TM-polarized reflected light and poles in thick layers 17
5.5 Poles for TE-polarization thick slabs 18
5.6 Discussion about poles and zeros in TM-polarization 18

1 Fourier plane microscopy and spectroscopy

Figure S1a shows the schema of the setup. Light from a Laser driven light source (LDLS) is collimated. The collimated light passes through a polarizer (excitation is TE polarized) before entering an inverted microscope (Nikon Eclipse TE2000-e), depicted here as the vertical items. In the microscope, light is redirected to the objective (60×, 1.49 NA, Nikon CFI Apo TIRF 60XC Oil, MRD01691) with a 50/50 beamsplitter (BS, Chroma 21000). The beam size has to be large enough to fill the back aperture of the objective in order to have information at the highest angles. Note that due to the high NA and the filling of the back aperture, the polarization of the field is no longer preserved at the sample, as shown in the inset of Figure S2a.

The reflected light (orange arrows) is focused into a Nikon D300s digital color camera or on a fiber coupled spectrometer. The real image of the sample (bright field) is produced with the objective and a lens. Instead, by replacing such lens with a Bertrand lens it is possible to image the back focal plane of the objective (showed in dotted red, also called Fourier plane). Pictures of the Fourier plane are taken with the color digital camera

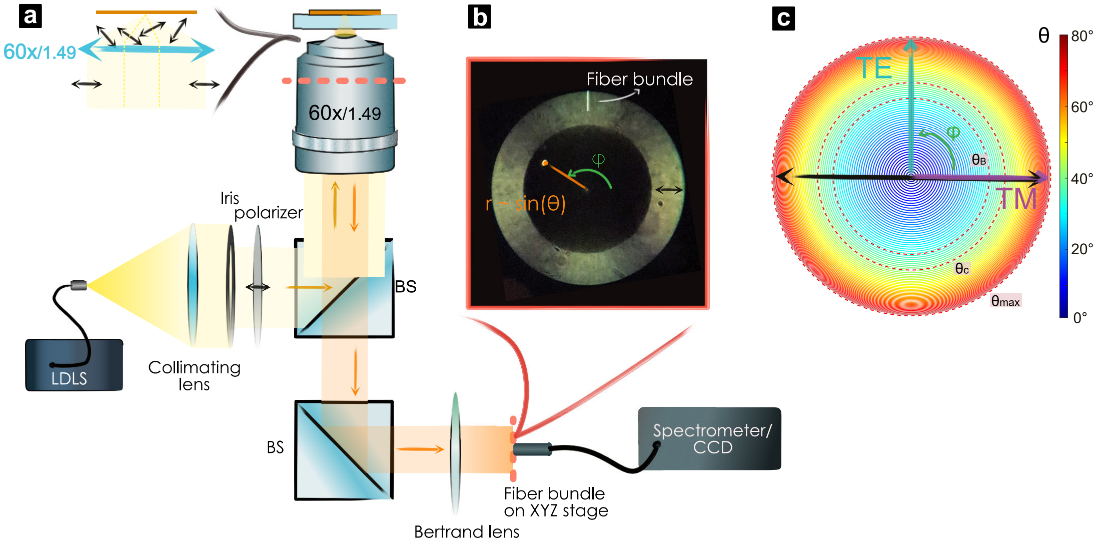

Figure S1: a) Fourier plane spectroscopy setup. White light is collimated, polarized (TE) and then focused on the sample through an oil immersion objective. Due to the high NA of the objective, the polarization is no longer preserved at the illumination spot (see black lines in inset). The Fourier plane of the objective is focused using a Bertrand lens, positioned before the microscope's image plane. Then the spectra is taken with a fiber bundle mounted on an XYZ stage. The Fourier plane is imaged with a digital camera. b) Digital color picture of the Fourier plane of bare glass. The fiber bundle is shown as a series of bright spots in a line. This is the detection for spectra. c) Angular distribution in the Fourier plane. This shows also the angle φ, that determines the distribution of TE and TM components in the Fourier plane.

(CCD). The spectroscopic information is obtained by using a Fiber bundle (19 fibers, 100 μm core, Andor SR-OPT-8002) coupled to a spectrometer (Andor Shamrock SR-500, equipped with a CCD detector Andor Newton 920).

The Fourier plane of bare glass (picture taken with a digital camera) is shown in Figure S1b. For angles above the glass/air critical angle $\theta > \theta_c = 41.5°$ there is total internal reflection. Therefore, in this region the lamp is perfectly reflected, thus we measured here the reference of the lamp for all angles. The fiber bundle was illuminated to make it visible in the same image. To cover all angles in the Fourier plane, the fiber bundle was mounted on a XYZ stage to control its position the Fourier plane.

The Fourier plane spreads the angular information along the radii as, $r \propto \sin(\theta)$ (shown in Figure S1c) and distributes the response to TE and TM polarized light. The intensity in the Fourier plane is given by

$$I(r,\varphi) \propto \frac{1}{cos(\theta)}(E_p E_p^* + E_s E_s^*) \tag{S1}$$

The TE (s) and TM (p) polarized components of the electric field in the Fourier plane have been calculated before [1]. In this case, all the signal is parallel to the sample, therefore the field components expressions reduce to

$$\begin{aligned} E_p &\propto c_1(\theta)\cos\theta\cos\varphi \\ E_s &\propto c_2(\theta)\sin\varphi \end{aligned} \tag{S2}$$

Where φ is the angle difference between the initial polarizer direction (polarization marked with a black line in Figure S1c) and the emitted light. From equation S2 it is clear that if $\varphi = 0°$, the field is purely TM polarized and that if $\varphi = 90°$ the field is purely TE. All other angles in between have a mix of both components. Therefore, the TE polarized measurements were taken along $\varphi = 90°$ and the TM ones along $\varphi = 0°$. Thus, the initial polarizer only rotates the orientation of the Fourier plane.

2 MoS_2 Thickness characterization

Monolayers of MoS_2 were identified by measuring their Photoluminescence (PL) [2] as shown in Figure S2b. Photoluminescence was measured in an upright microscope (Nikon Eclipse LV150N) with a 100× objective (100× Nikon CFI60 TU Plan Epi ELWD). The broadband excitation lamp (CoolLED pE-300) was filtered with a bandpass of 400-488 nm. The measured power after the 100× objective was 49.2 mW. The collection spot was 1.8 μm in diameter. Once the monolayers were identified, the samples were characterized with Atomic Force Microscopy (AFM) and optical contrast. The monolayers were used as a baseline to characterize the height in both cases.

The thickness was also characterized by optical contrast [3]. The AFM measurements are challenging because the glass substrates are rough, so to avoid potential errors by AFM measurements we checked the thickness with a second method, optical contrast. Optical contrast is the difference of the intensity of light reflected by a flake and the substrate,

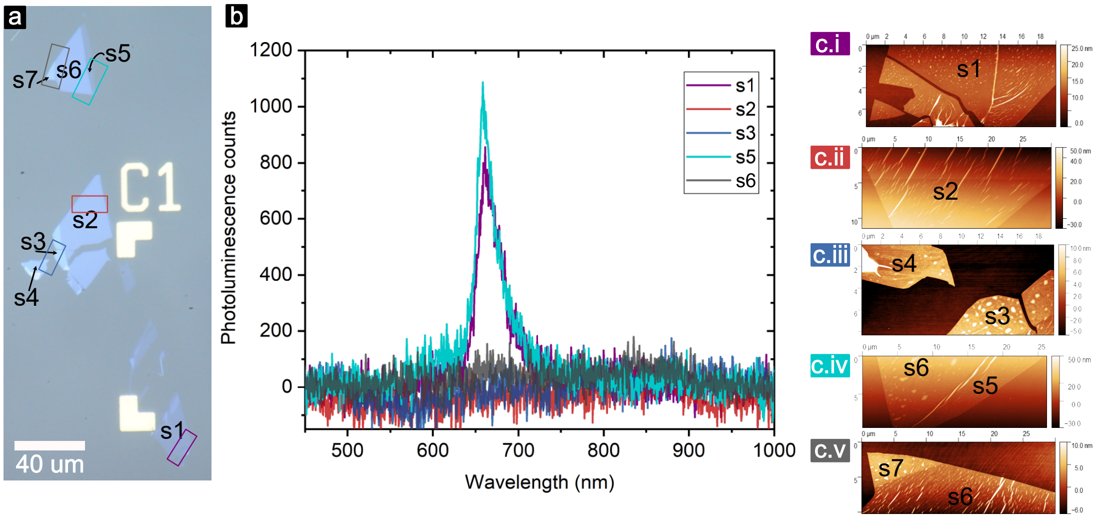

Figure S2: Monolayer thickness characterization of thick layers a) Bright field image in reflection. b) Photoluminescence counts upon 488 nm illumination. c) Atomic Force Microscopy (AFM) images for thickness characterization of the samples from which the following thicknesses were averaged i) Monolayer, s1, $d = 0.7 \pm 0.2$ ii) s2, $d = 1.5 \pm 0.6$ nm iii) s3, $d = 3.7 \pm 0.4$ nm and s4, $d = 5.4 \pm 0.3$ nm iv) s5, $d = 0.6 \pm 0.2$, and s6, $d = 1.6 \pm 0.4$ and v) s6 and s7 $d = 3.7 \pm 0.4$. Note that the AFM images are rotated with respect to a).

which is then normalized by the intensity reflected from a monolayer (identified by PL measurements as mentioned above). The normalized counts are shown in Figure S3. The analysis was done in ImageJ using profile analysis along the lines marked in colors on the bright field image in Figure S3a. We obtain the grey values (brightness of a pixel) by plotting the profile along the given line. We subtract the grey value of the background from the monolayers (Figure S3c,e) and we use such value to normalize all other profiles. Therefore, we obtain the number of layers via the normalizing the contrast profiles, these are shown in Figure S3b-e. In general, both AFM and optical contrast agree.

Figure S4 shows AFM measurements for the rest of the layers used in this study. Their thickness is shown in the caption. From a-c it shows the samples in the thin regime, discussed in section II in the main text. The thick flakes shown in S4e were used in section III in the main text. Most of the slabs in S4a-d were used for data presented in this supporting information.

3 Reflection and absorption calculations

3.1 Permittivity for calculations

Reflection calculations were done by TMM [5] (see methods). The experimental permittivity of MoS_2 was fitted as a Lorentz material with 3 resonances given by the A, B and

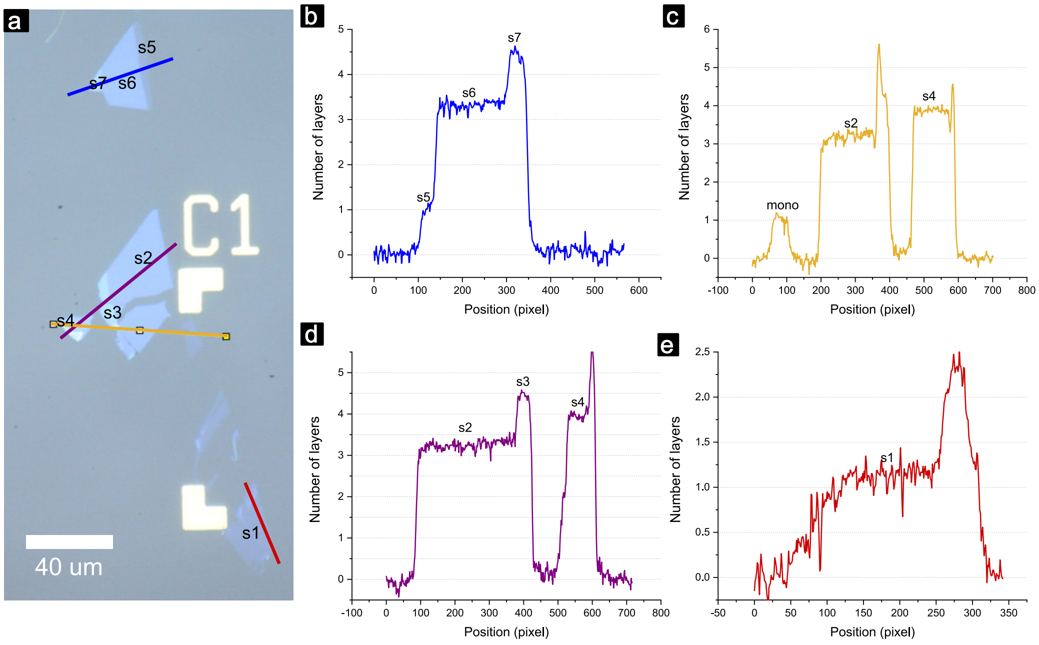

Figure S3: Optical contrast of thin MoS_2 flakes. a) Bright field image with the scanned area in colored lines. b) The blue line goes through s5 (1L), s6 (3L) and s7 (4-5L). c) The yellow line goes through a monolayer, s2(3L) and s4 (4L). d) The purple line goes through s2 (3L), s3 (4-5L) and s4 (4L). e) The red line goes through s1 (1L).

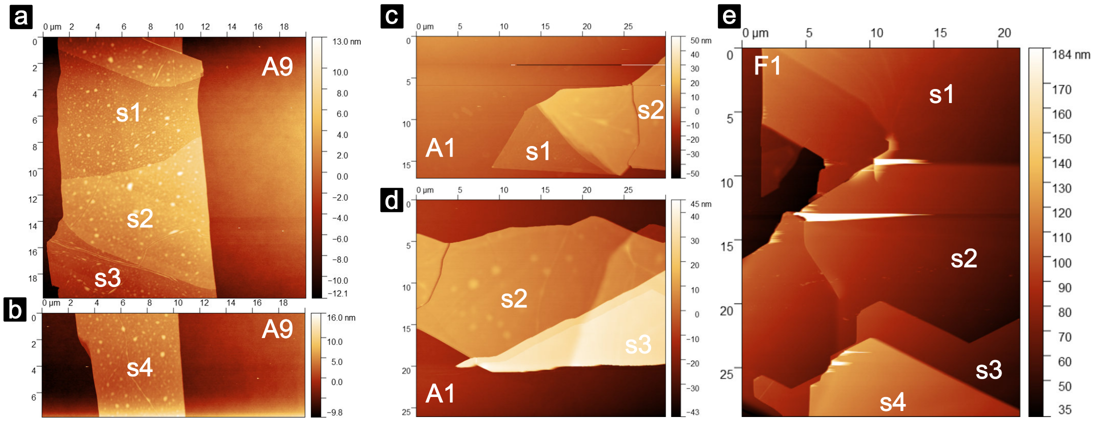

Figure S4: AFM images for thickness characterization of other layers a) Sample A9 s1 $d = 2.6 \pm 0.6$ nm, s2 $d = 4.6 \pm 0.4$ nm. s3 $d = 4.0 \pm 0.3$ nm b) A9 s4 $d = 4.9 \pm 0.4$ nm c) A1 s1 $d = 3.9 \pm 0.5$ nm, s2 $d = 21.0 \pm 0.7$ nm d) A1 s2 and s3 $d = 58.8 \pm 0.5$ nm e) F1 s1 $d = 48 \pm 1$, s2 $d = 67 \pm 2$ nm, s3 $d = 87 \pm 2$ nm and s4 $d = 125 \pm 2$ nm.

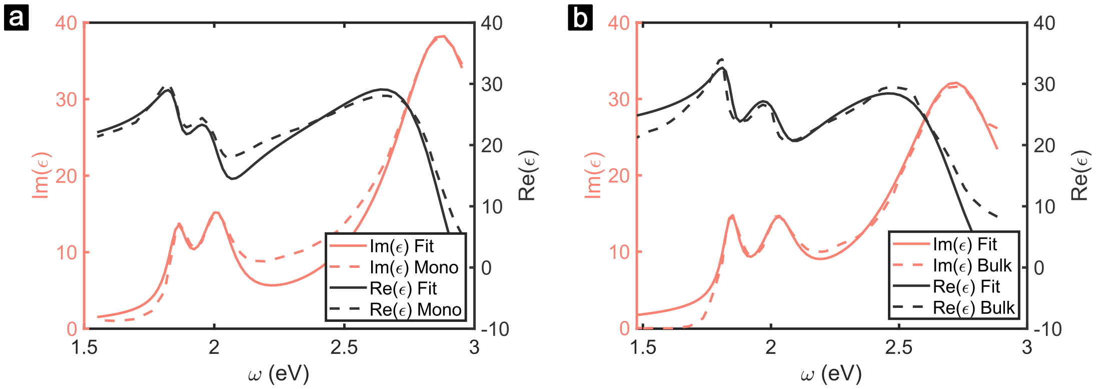

Figure S5: Experimental data of permittivity of MoS_2 (dashed lines) [4] and the permittivity fitted with Lorentzians (solid lines). (a) Data for a monolayer. (b) Data for MoS_2 in bulk.

C excitons to fit the experimental permittivity [4]:

$$\varepsilon(\omega) = \varepsilon_\infty + \sum_{i=1}^{3} f_i \frac{\omega_{P,i}^2}{\omega_{0,i}^2 - \omega^2 - i\gamma_i\omega} \tag{S3}$$

Where for 3L and more, we used the bulk permittivity. The constants used for the bulk permittivity fitting were: $\varepsilon_\infty = 13$, $\omega_{0,1} = 1.845$ eV, $\gamma_{0,1} = 0.08$ eV, $f_1\omega_{P,1}^2 = 1.53$ eV^2, $\omega_{0,2} = 2.03$ eV, $\gamma_{0,2} = 0.17$ eV, $f_2\omega_{P,2}^2 = 3.38$ eV^2, $\omega_{0,3} = 2.73$ eV, $\gamma_{0,3} = 0.55$ eV, $f_3\omega_{P,3}^2 = 47.92$ eV^2. The fitting is shown in Figure S5b.

For a monolayer and bilayer the constants used for the fitting were: $\varepsilon_\infty = 10$, $\omega_{0,1} = 1.86$ eV, $\gamma_{0,1} = 0.09$ eV, $f_1\omega_{P,1}^2 = 1.56$ eV^2, $\omega_{0,2} = 2.01$ eV, $\gamma_{0,2} = 0.15$ eV, $f_2\omega_{P,2}^2 = 3.64$ eV^2, $\omega_{0,3} = 2.88$ eV, $\gamma_{0,3} = 0.46$ eV, $f_3\omega_{P,3}^2 = 50.59$ eV^2. The fitting is shown in Figure S5a.

3.2 Monolayer TM absorption

The absorption of the monolayer was described in the main text for TE- polarization. In Figure S6 we show the calculation of the TM-polarization absorption of a monolayer. Figure S6a shows the maximum of absorption (in the visible) for various angles. Interestingly, the minimum of reflection (in all the visible) reaches 100% at the critical angle. Meaning that at the critical angle, most of the light reflected by a monolayer is TM-polarized. Moreover in Figure S6b we can see that at θ_c the absorption is 0 for all wavelengths (blue line). Thus, the TM light is perfectly reflected at θ_c for all wavelengths. For TE, reflection at θ_c reaches a maximum of 97% only for energies below the A-exciton (Figure 2c). Regarding absorption, the maximum (C-exciton at 55°) only reaches 35%, thus the enhancement is of 1.34× at the C-exciton with respect to normal incidence.

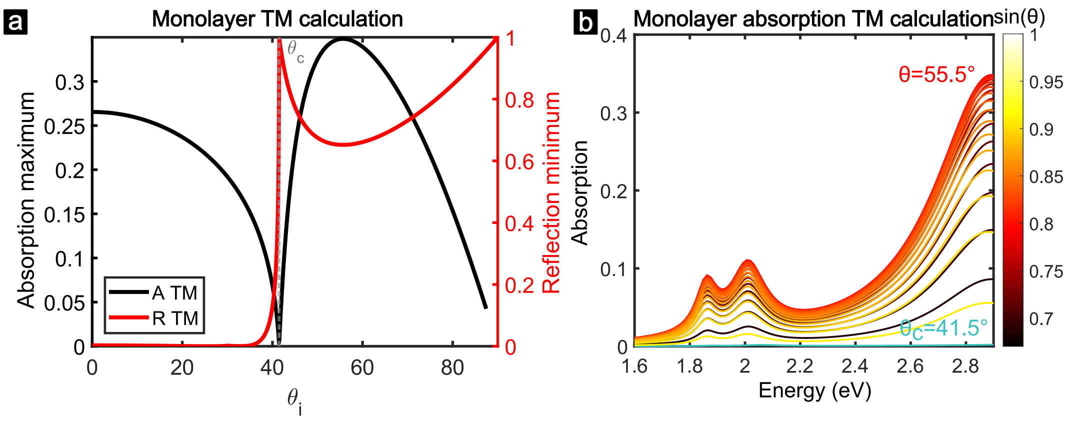

Figure S6: a) Monolayer absorption maximum and reflection minimum in the visible (1.6-2.9 eV) for all angles. Calculation for TM-polarization. The dotted line marks the critical angle, θ_c. b) Calculation of absorption spectra of TM-polarization in the visible at various angles beyond θ_c. The color is given by $\sin\theta$.

4 Angular spectral sensitivity in thin layers of MoS_2

4.1 Calculation of angular reflection spectra of TE-polarized light

As described in section III of the main text, thin MoS_2 layers absorb light in very different frequencies and angles. The difference is outstanding for a single-layer increase in thickness. In the main text (Figure 3) we saw the different spectra for only 3 thicknesses. In Figure S7 we show the calculations of angular spectra for slabs with 1L - 9L. The difference is clear on a single-layer level, thus making it an unambiguous technique to distinguish the thickness in thin slabs below 10L. Above that threshold all the angular spectra start reflecting most of the TE light and there is slight absorption only at the frequency of the excitons.

4.2 Experimental angular reflection spectra of TE-polarized light

The experimental equivalent of Figure S7 is shown in Figure S8. All the data presented in this figure is from different flakes than showed in Figure 3. Thus the angular spectra are reproducible for similar thickness flakes. In theory we showed that the thicknesses in panels c-f present perfect absorption. As mentioned above, noise in the experimental setup limits the measurable reflection to a minimum of 10^{-2}.

Figure S8c is clearly a 4L flake as shown in optical contrast (Figure S3) and by comparing with theory (Figure S7). Nevertheless the AFM measurements show a larger thickness. This is most likely related to the small size of the flake and to the big bubble in it (Figure S2c.iii).

Smaller differences between experiments and calculations may be given by the anisotropy in MoS_2 permittivity, which was not considered for the calculations (see Methods and SI

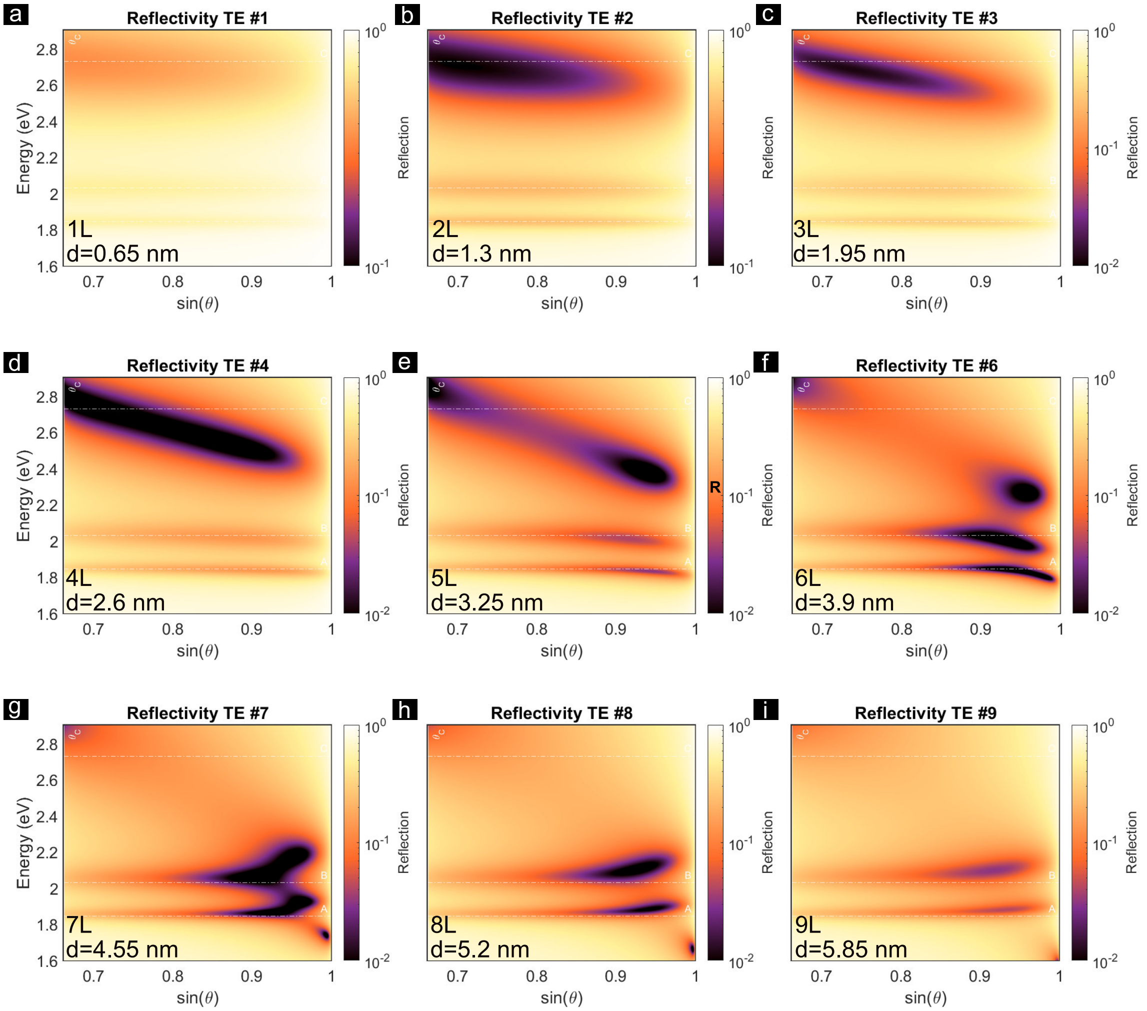

Figure S7: Calculation of angular spectral sensitivity to thickness in thin MoS_2 slabs. Calculation of reflection beyond θ_c with TMM for TE-polarized light. Each layer is considered to be 0.65 nm. Flakes with 4L - 8L (panels d-h) show perfect absorption. The resolution in reflection was limited to 10^{-2} to match the experimental limit. Note the difference in the reflection colorbar limits for a and b, where the minimum is 10^{-1} instead.

sec. 4.4).

4.3 $\hat{S}$-matrix zeros in thin MoS_2 layers

The differences in reflection angular spectra by varying the thickness can be explained by finding the positions of the zeros of the S-matrix in the complex-ω plane.

Figure S9ii shows the zeros for different angles beyond θ_c. When the imaginary part of the frequency of the zero vanishes, they are fully real and there is perfect absorption. To see the correlation, Figure S9 shows the zeros positions in the complex frequency

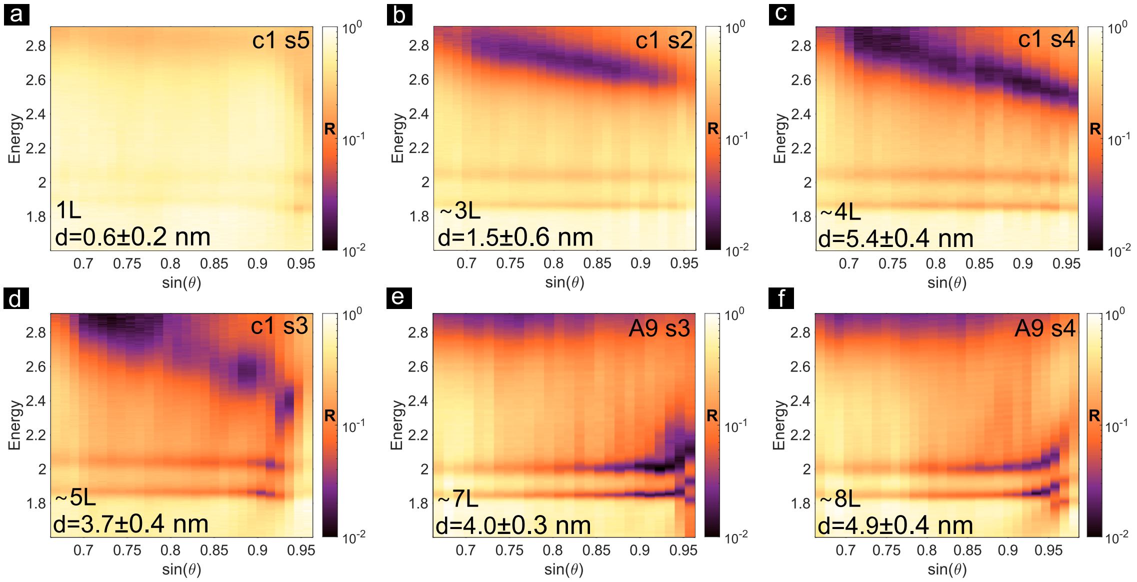

Figure S8: Experiment of angular spectral sensitivity to thickness in thin MoS_2 slabs. Measurements of reflection of TE polarized light. The thickness was measured by AFM a) Monolayer $d = 0.6 \pm 0.2$ nm, b) around 3L, $d = 1.5 \pm 0.6$ nm, c) approx. 4L from the optical contrast measurement, d) around 5L $d = 3.7 \pm 0.4$ nm, e) around 6-7L $d = 4.0 \pm 0.3$ nm, from comparison to calculation it should be 7L f) around 8L $d = 4.0 \pm 0.3$ nm.

plane and the phase, ϕ. As was mentioned in the main text, ϕ, $E_{ref}/E_{inc} = |r|e^{i\phi}$, has a singularity when the reflected field is zero, $E_{ref} = 0$. Moreover, beyond the critical angle T=0, thus the singularity appears when the absorption is perfect.

Due to the high loss of the excitons, the zeros of the $\hat{S}$-matrix appear in the lower half of the complex plane for thin slabs (Figure S9a), thus there are no singularities. Increasing the thickness pulls the zeros to the upper half of the complex plane. Due to its high oscillator strength (table 1), the first zeros to cross the real axis are the ones given by the C-exciton for 5L (Figure S9b).

In general singularities come in pairs for thin slabs, as observed before for metasurfaces [6]. There is one pair per excitonic resonance (Figure S9) and they have opposite topological charge. Even though they are related to the same resonance, the pairs appear at different angles and frequencies because the 3 zeros (one zero related to each exciton per angle) make a loop trajectory around the exciton when varying the angle of incidence (Figure S9c). Thus they cross the real frequency axis at different angles and frequencies. All of them appear at different angles and frequencies. In Figure S9c we can see an example close to this situation, where for a 7L slab there are 5 singularities. Four of those are given by A and B excitons, the last one is given by the C-exciton.

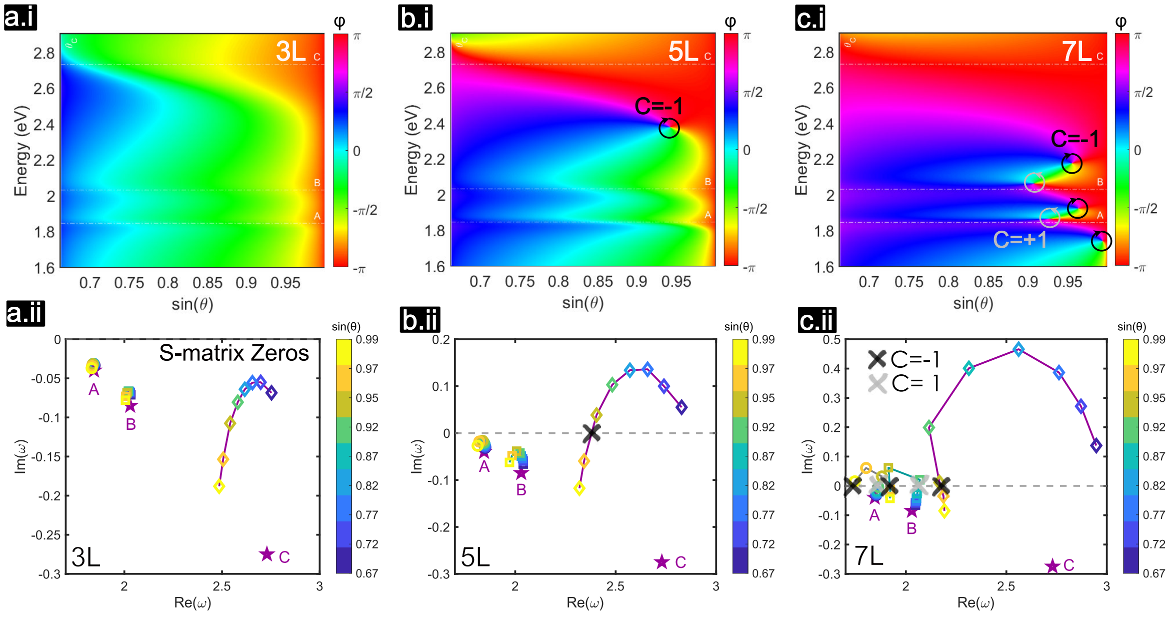

Figure S9: (i) Phase (ϕ), $E_{ref}/E_{inc} = |r|e^{i\phi}$, for MoS_2 slabs different thicknesses: (a) 3 layers with $d = 1.2$ nm, (b) 5 layers with $d = 3.25$ nm, and (c) 7 layers with $d = 4.55$ nm. (ii) Zeros of the $\hat{S}$-matrix positions in the complex-ω plane at different angles of incidence $\sin\theta$ for the same thicknesses as above.

4.4 Direct calculation of perfect absorption condition for thin films

The thickness and angle for which there will be perfect absorption of TE and TM-polarized light can be calculated from Fresnel equations. We consider an Attenuated total internal reflection situation as in the schema of Figure S10a. Here the incident media has a higher permittivity than the outgoing media, $\varepsilon_1 > \varepsilon_3$, and the permittivity of the layer $\varepsilon = \Re(\varepsilon) + i\Im(\varepsilon)$ is higher than both. TIR occurs for angles such that $\theta > \theta_c = \arcsin\frac{n_3}{n_1}$. In this case,

$$\begin{aligned} k_1 &= k_0\sqrt{\varepsilon_1}\cos\theta \\ k_2 &= k_0\sqrt{\Re\varepsilon + i\Im\varepsilon - \varepsilon_1\sin^2\theta} \\ k_3 &= k_0\sqrt{\varepsilon_1\sin^2\theta - \varepsilon_3} \end{aligned} \tag{S4}$$

Where k_3 represents an evanescent wave and $k_0 = \omega/c$.

The reflection coefficient from the flake is given by

$$r = \frac{r_{12} + r_{23}e^{2ik_2d}}{1 + r_{12}r_{23}e^{2ik_2d}}\ . \tag{S5}$$

Then perfect absorption occurs when

$$r_{12} + r_{23}e^{2ik_2d} = 0\ . \tag{S6}$$

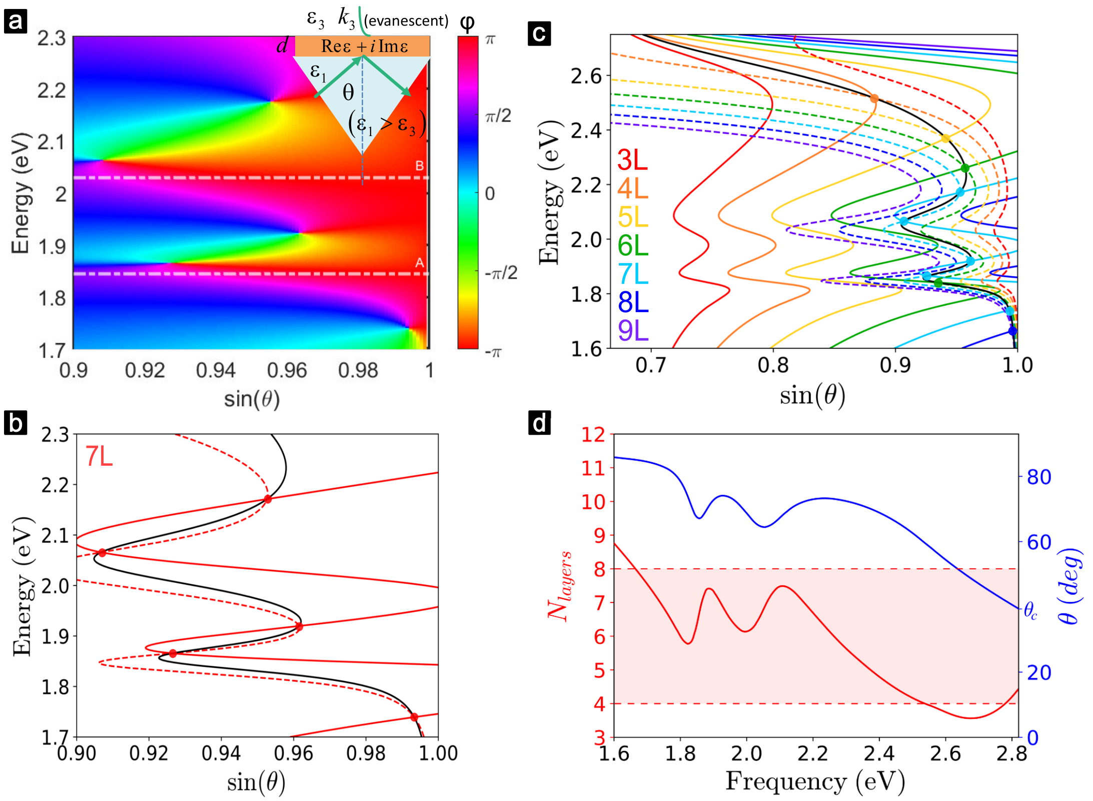

Figure S10: Analytical calculation of the perfect absorption condition in thin MoS_2 films for TE-polarization. a) Phase of the reflected wave in a 7L MoS_2 slab. The singularities correspond to the perfect absorption points. The inset shows a schema of the analytical calculation in TIR configuration. b) Perfect absorption condition on $\Re\varepsilon$ (solid red line) and $\Im\varepsilon$ (red dashed line) for a 7L MoS_2 layer. The intersection between solid and dashed lines denote the frequency and angles at which there is perfect absorption (marked in red circles) which correspond to the singularities in the phase in a). c) The same as in (b) for thicknesses of 3L-9L (violet - red). The perfect absorption points are the intersections between the solid and dashed lines and are marked with colored circles for each thickness. All circles lie on the black line, which shows the frequencies and angles at which there can be a singularity for all possible thicknesses. d) Frequency dependencies of all possible angles and thicknesses satisfying the perfect absorption conditions.

Using the Fresnel coefficients for TE waves

$$r_{12} = \frac{k_1 - k_2}{k_1 + k_2}\ ,\ r_{23} = \frac{k_2 - ik_3}{k_2 + ik_3}\ , \tag{S7}$$

we find that the condition for perfect absorption of TE-polarized light is

$$\tanh\left(ik_2 d\right) = \frac{k_2(k_1 - ik_3)}{ik_1k_3 - k_2^2}\ . \tag{S8}$$

This means that the thickness for which there is perfect absorption in the thin layer is

$$d = \frac{1}{k_2}\Im\Big(\tanh^{-1}\left[\frac{k_2(k_1 - ik_3)}{ik_1k_3 - k_2^2}\right]\Big) . \tag{S9}$$

For thin films we can consider $k_2d \ll 1$ and if $\varepsilon_{1,3} \ll \varepsilon$ then $k_1k_2 \ll k_2^2$. Then equation S8 can be approximated to

$$ik_2^2 d \approx (ik_3 - k_1) . \tag{S10}$$

By using that $k_2^2 = k_0^2\left(\Re\varepsilon + i\Im\varepsilon - \varepsilon_1 \sin^2\theta\right)$, equation S10 can be rewritten as

$$ik_0^2\left(\Re\varepsilon + i\Im\varepsilon - \varepsilon_1 \sin^2\theta\right) \approx \frac{(ik_3 - k_1)}{d} . \tag{S11}$$

This equation can be separated in real and imaginary part, giving rise to a pair of equations that must be fulfilled simultaneously to find perfect absorption.

$$\Re\varepsilon = \varepsilon_1 \sin^2\theta + \frac{k_3}{dk_0^2} , \tag{S12}$$

$$\Im\varepsilon = \frac{k_1}{dk_0^2} . \tag{S13}$$

This equations allow to obtain the thicknesses of slabs, frequencies and angles at which there will be perfect absorption for a material with a given permittivity in TIR configuration.

To find a connection between all possible angles and frequencies at which there could be perfect absorption, for arbitrary thicknesses, it is convenient to combine equations S12 and S13 by getting rid of d:

$$\Im\varepsilon = \sqrt{\frac{\varepsilon_1 \cos^2\theta}{\varepsilon_1 \sin^2\theta - \varepsilon_3}}\left(\Re\varepsilon - \varepsilon_1 \sin^2\theta\right) . \tag{S14}$$

From this equation, one can find the general relationship between the angle and the components of the permittivity, which for given $\varepsilon(\omega)$ yields the frequency dependence of the desired angles $\theta(\omega)$. Then, substituting this dependence to the equation S12 or S13, one obtains the frequency dependence of the suitable thicknesses $d(\omega)$. The experimental limitation on the maximum observed angle on one side and the critical angle of total internal reflection on the other side gives a narrow window of allowable for the perfect absorption number of layers.

Figure S10b shows the plots of equations S12 and S13 for MoS_2 for 7L. The intersection of both red lines (dashed and solid) are highlighted with circles and represent the points of perfect absorption in frequency and angles. Those points match to the singularities in the phase of the reflected wave (Figure S10a). Figure S10c shows the same as S10b for thicknesses between 3L-9L (violet-red lines). The intersections of dashed and solid

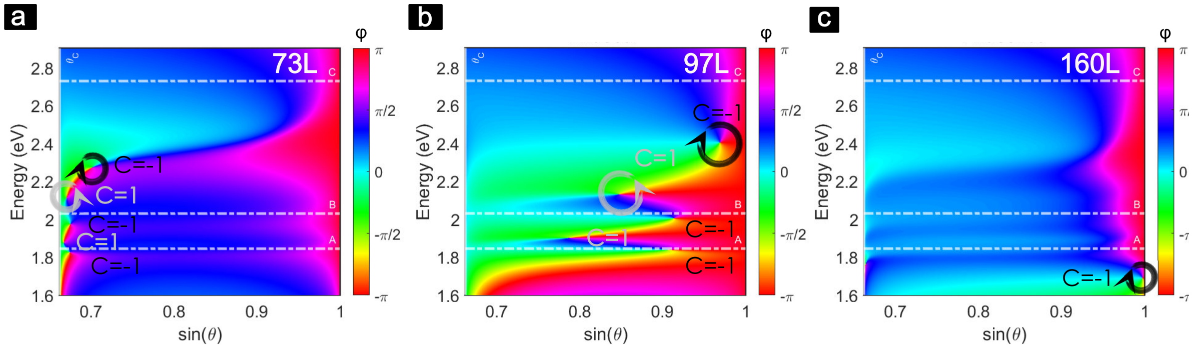

Figure S11: Phase of the reflected TM polarized light (ϕ), $E_{ref}/E_{inc} = |r|e^{i\phi}$, for MoS_2 slabs different thicknesses: (a) 73L, (b) 97L and (c) 160L. The singularities have both topological charges marked in black $C = -1$ and grey $C = 1$.

lines for a fixed thickness are marked with circles of corresponding color. Here is visible that intersections are only possible for 4L-8L in the studied spectral window (1.6 -2.9 eV). Before and after there are no intersections, and thus no perfect absorption. All the intersections of dashed and solid lines in Figures S10b,c lie on the black line which shows angles and frequencies for arbitrary thicknesses at which there could be perfect absorption (see equation S11). The frequency dependencies of the angles $\theta(\omega)$ and thicknesses $d(\omega)$ obtained from the equations S14 and S13 are shown in Figure S10d.

The same approach can be used for TM-polarized light. Using the corresponding Fresnel coefficients,

$$r_{12} = \frac{k_1/\varepsilon_1 - k_2/\varepsilon_2}{k_1/\varepsilon_1 + k_2/\varepsilon_2} \;, \; r_{23} = \frac{k_2/\varepsilon_2 - ik_3/\varepsilon_3}{k_2/\varepsilon_2 + ik_3/\varepsilon_3} \;. \tag{S15}$$

Then we find that the analogous of equation S9 for TM is

$$d = \frac{1}{k_2}\Im\Big(\tanh^{-1}\left[\frac{k_2\varepsilon_2(k_1\varepsilon_3 - ik_3\varepsilon_1)}{ik_1k_3\varepsilon_2^2 - k_2^2\varepsilon_1\varepsilon_3}\right]\Big) \;. \tag{S16}$$

5 Reflection of thick slabs (d>10L)

5.1 Phase singularities in thick layers of TM-polarized light

Figure 4 in the main text showed that thick slabs of MoS_2 sustain Fabry-Pérot modes that give beautiful colors to the reflection of the slabs. Also the angular spectra of reflected TM-polarized light showed great absorption in bands. From this figure it is not clear which points show perfect absorption. Thus, Figure S11 shows the phase for the same thicknesses. The singularities mark precisely the points of perfect absorption. Their topological charge is also marked in grey $C = 1$ and black $C = -1$.

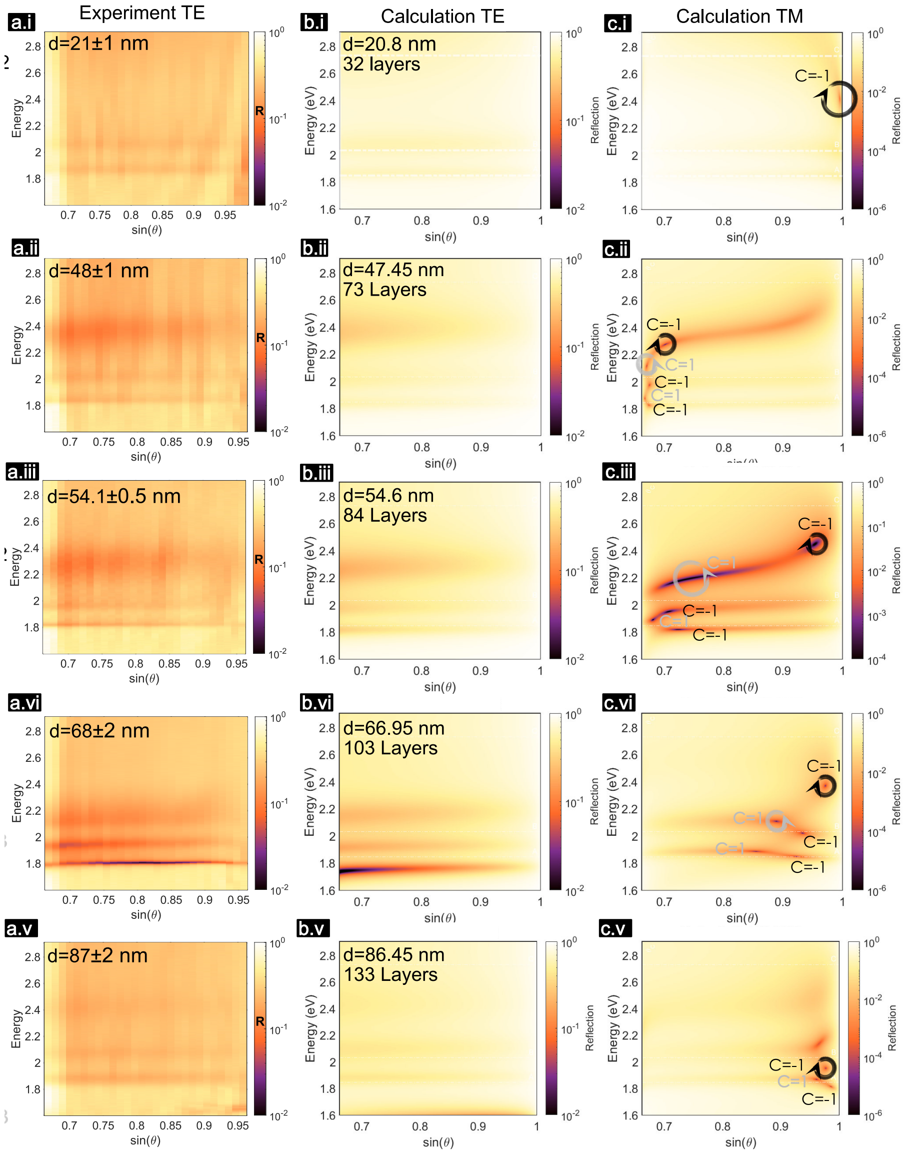

Figure S12: Angle-dependent reflection spectra from thick MoS_2 slabs for various thicknesses around i) 32L, ii) 73L, iii) 84L, iv) 103L and v) 133L. a) Measurements of reflection of TE-polarized light for various increasing thicknesses (i-v). b) Calculation of the TE-polarized light reflection spectra matching the sale thicknesses. None of the thicknesses show perfect absorption. c) Calculation of the TM-polarized light reflection spectra for the same thicknesses. There is perfect absorption for all the thicknesses, giving rise to a singularity with topological charge shown in black $C = -1$ and in grey $C = 1$. Note the difference in the logarithmic scale of the colormap, whose minimum is 10^{-6}.

5.2 Reflection of TE-polarized light by thick layers

Figure S12a shows the experimental angle-dependent reflection spectra for TE-polarized light of various thicknesses, $d > 10$L. The calculation for the same thicknesses is shown in

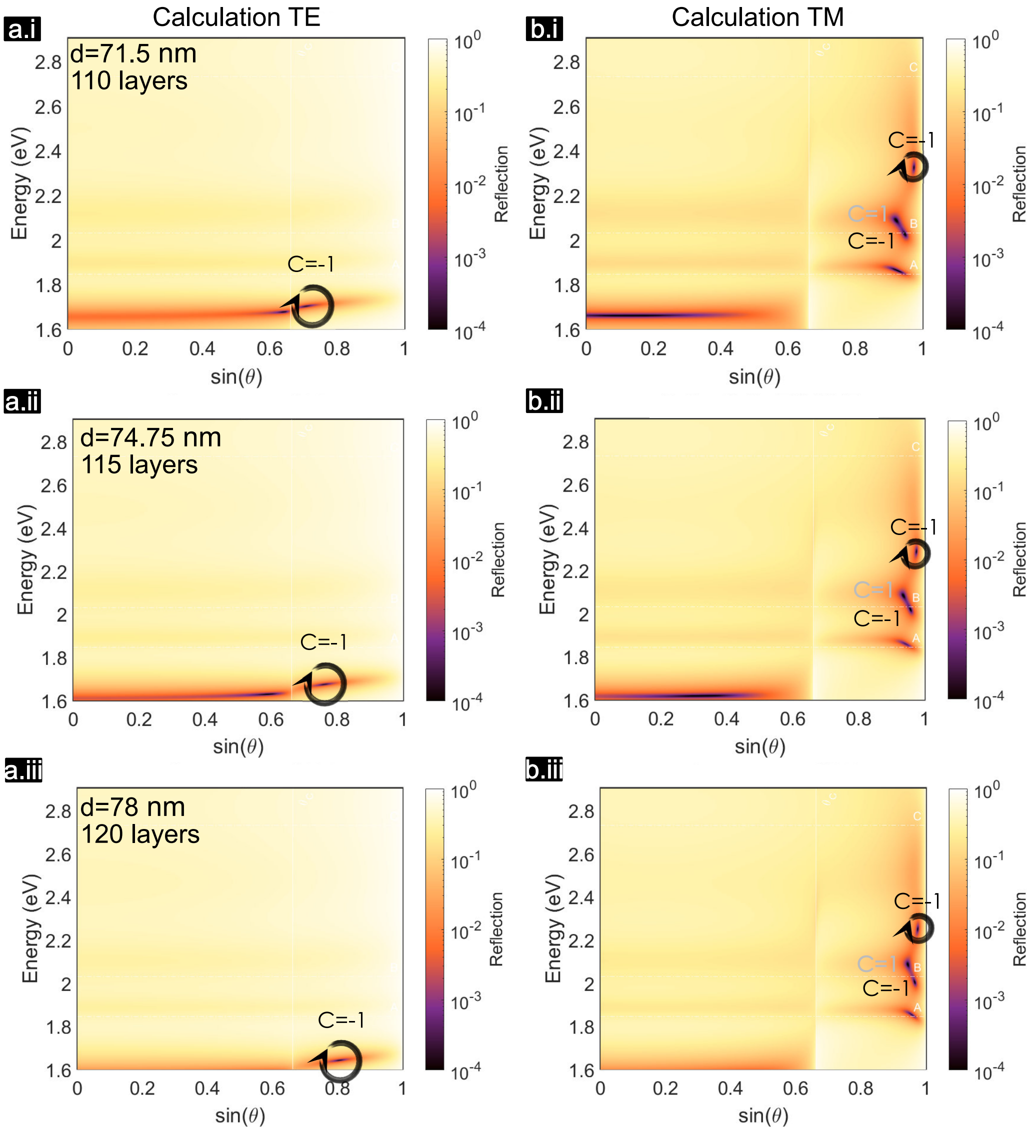

Figure S13: Window of thicknesses for which both polarizations are perfectly absorbed by the same flake i)110L, ii) 115L and iii) 120L. a) Calculation of TE-polarized reflected light showing one point of perfect absorption giving rise to a singularity with topological charge of $C = -1$. b) Calculation of TM-polarized reflected light showing 3 points of perfect absorption which give rise to singularities of both topological charges as marked in black, $C = -1$, and grey, $C = 1$.

Figure S12b. The self-sustained optical mode below the light-line, due to the thickness of the slab, appears as a clear minimum in reflection in S12ii,iii. Increasing the thickness red-shifts the mode until it starts interacting with the excitons as in Figure S12iv. Increasing the thickness even further causes a higher order optical mode to appear as in Figure S12v.

TE-polarization does not show any points of perfect absorption in Figure S12b, but for the same thicknesses they appear for TM (Figure S12c). Depending on the thickness

there can be up to 5 points of perfect absorption with opposite optical charges. The topological charge of each singularity is marked in black, $C = -1$ or grey $C = 1$.

5.3 Perfect absorption of both TE and TM-polarized light in the same MoS_2 slab

Figure S13 shows some examples of the small window of thicknesses (110L-120L) at which there is perfect absorption for both polarizations. For these thicknesses perfect absorption occurs in the same structure but at different angles and photon energies. The points of perfect absorption are marked in both cases with wither circles or the label of the topological charge of the singularity.

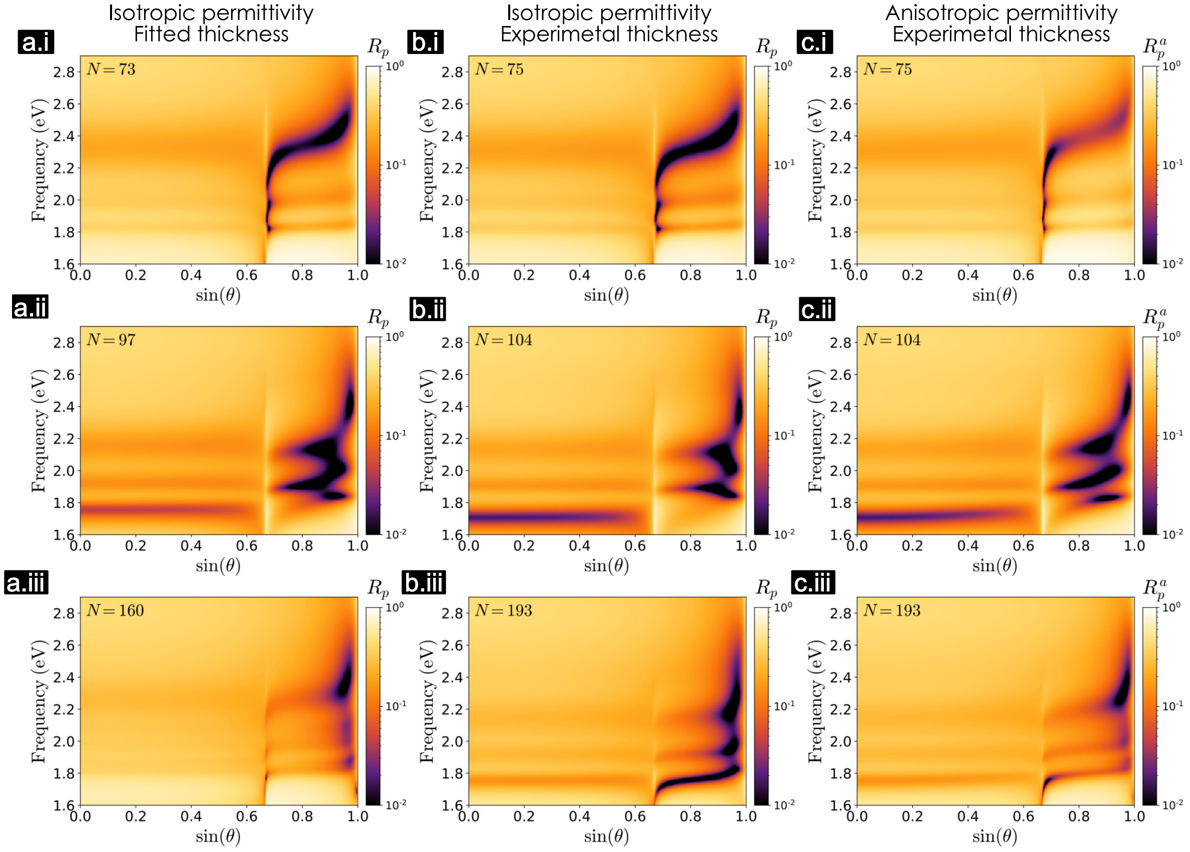

Figure S14: Comparison between calculation of reflection of TM-polarized light considering MoS_2 as isotropic and anisotropic. a) Isotropic calculations considering the thickness that gave the closer similarity to the experimental angular spectra (Same as in Figure 4). The thicknesses are i) $d = 47.45$ nm, ii) $d = 63$ nm, and iii) $d = 104$ nm. b) Isotropic calculation considering the thickness measured by AFM (Figure S4). The thicknesses are i) $d = 48.75$ nm, ii) $d = 67.6$ nm, and iii) $d = 125.45$ nm c) Anisotropic calculation considering the thickness measured by AFM (Figure S4). The thicknesses are i) $d = 48.75$ nm, ii) $d = 67.6$ nm, and iii) $d = 125.45$ nm.

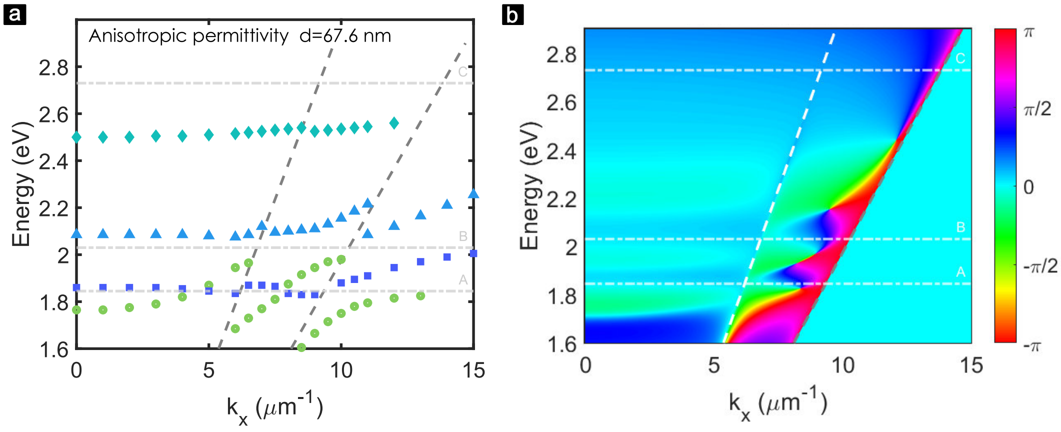

Figure S15: Anisotropic calculations for TM-polarized light showing strong coupling and polaritons. a) Dispersion of poles of $\hat{S}$-matrix of considering an anisotropic flake of 104L $d = 67.6$ nm. The B-exciton shows anti-crossing, therefore is strongly coupled both above and below the air light-line. b) Phase of the reflected TM-polarized light for the same anisotropic flake. It shows the singularities related to perfect absorption.

5.4 Anisotropic MoS_2 calculation of TM-polarized reflected light and poles in thick layers

2D TMDs are highly anisotropic and MoS_2 is no exception [7]. Anisotropy is particularly important for TM-polarization because the electric field is not solely in-plane. To take anisotropy into account one needs to make a standard substitution in the reflection coefficients S15 and S5: $k_2 \to k_0\sqrt{\varepsilon_\parallel/\varepsilon_\perp}\sqrt{\varepsilon_\perp - \varepsilon_1 \sin^2\theta}$ and $\varepsilon_2 \to \varepsilon_\parallel$. In Figure S14 the isotropic and anisotropic cases are compared. In this case $n_\perp = 2.74$ [7] and $\varepsilon_\parallel$ was considered to be the same as given by the fitted Lorentzians in equation 3 in the main text, we also tried with the data in [7] and the changes are minimal (not showed here). Taking into account anisotropy provides a better correspondence with the experimental number of layers (see Figure 4 in the main text).

Additionally, Figure S15a shows that the poles in the anisotropic case have the same behavior as the data presented in Figure 5. The B-exciton is strongly coupled above and below the light-line, whereas the A-exciton is not. Moreover, the Rabi splitting of the B-exciton with the photonic mode above the light-line is $\Omega_{R_I} = 0.12$ eV and below the air light-line is $\Omega_{R_I I} = 0.16$ eV, which is larger than the values presented in Figure 5 considering an isotropic flake.

Moreover, Figure S15b shows the singularities related to perfect absorption in the same flake. Therefore, the anisotropy does not change the general behavior described in the main text.

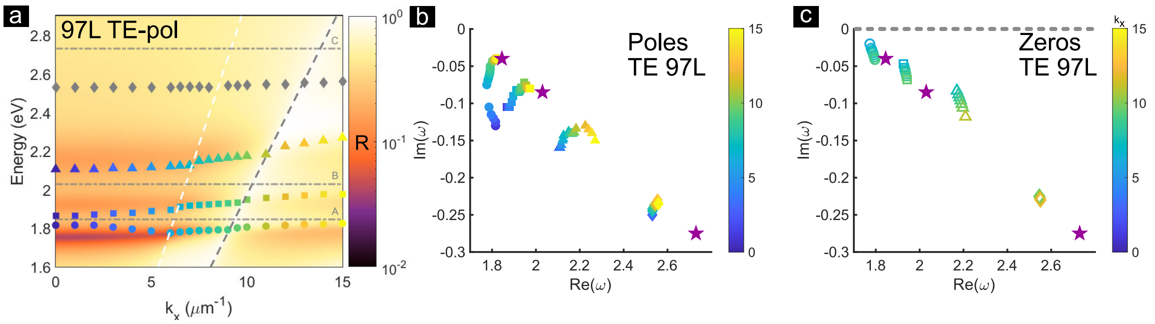

Figure S16: a) Calculated dispersion of the TE-polarized reflection 97L TE modes. b) Trajectories of the poles in the complex$-\omega$ plane for various wave vectors, k_x in different colors. c) Zeros trajectories in the complex$-\omega$ plane for different k_x. All the zeros appear in the lower half of the complex plane, meaning that there are no points with perfect absorption.

5.5 Poles for TE-polarization thick slabs

Figure 5 in the main text shows the calculated dispersion $\hbar\omega$ vs $k_x = n\,k_0\,\sin\theta$ of the reflection of TM-polarized light for 97L of MoS_2 with the poles of the eigenmodes of the $\hat{S}$-matrix (QNMs) plotted on top. Figure S16 shows the same information for 97L for TE-polarized light.

Note that the three regions mentioned in the main text appear again. Region I (above the LL) is a two-port system because it can be illuminated from both sides of the flake. On the contrary, region II (beyond air LL) is limited to one-port because radiation happens solely through glass. In this region, perfect absorption can take place. In two-port systems, a similar concept to perfect absorption can be obtained, this is coherent perfect absorption where the phase has to be matched [8, 9, 10]. This increases the challenge of fabrication and design of the input of light in the samples.

In the TE-polarized light case, there are no perfect absorption points, since all the zeros are below the real axis.

5.6 Discussion about poles and zeros in TM-polarization

As mentioned in the main text, Figure 5d shows that two singularities can arise from the interaction with a single exciton because the zero is pulled towards the exciton (giving rise to a $C = -1$ topological charge) and then goes back to the upper half (giving rise to a $C = 1$ singularity) until the next exciton pulls it to the lower half of the complex plane. This gives rise to perfect absorption points because of the interplay between the photonic and the excitonic modes even if the coupling is not strong. This remark is interesting because, contrary to our case, phase singularities appearances have been related to strong coupling above the LL in organic molecules [11].

Also, the polaritonic eigenmodes do not perfectly absorb light. In order for them to happen a zero should occur at the same frequency and angle as a pole. It has been

theorized before that the frequencies of the zeros and poles in a strongly coupled system, in a two-port symmetric system, would be the same only if the radiative losses of the cavity would be zero [9], which is not possible at all in a lossy slab. Actually, in such two-port systems, coherent perfect absorption and polaritons have been observed simultaneously only thanks to careful design and fabrication [12].

References

[1] Lieb, M. A., Zavislan, J. M. & Novotny, L. Single-molecule orientations determined by direct emission pattern imaging. *J. Opt. Soc. Am. B* **21**, 1210–1215 (2004).

[2] Splendiani, A. *et al.* Emerging photoluminescence in monolayer mos_2. *Nano Lett.* **10**, 1271–1275 (2010).

[3] Li, X.-L. *et al.* Layer-number nependent optical properties of 2d materials and their application for thickness determination. *Adv. Funct. Mater.* **27**, 1604468 (2017).

[4] Li, Y. *et al.* Measurement of the Optical Dielectric Function of Monolayer Transition-Metal Dichalcogenides. *Phys. Rev. B* **90**, 205422 (2014).

[5] Born, M. & Wolf, E. Principles of optics, seventh (expanded) edition. *Cambridge U. Press, Cambridge, UK* (1999).

[6] Berkhout, A. & Koenderink, A. F. Perfect absorption and phase singularities in plasmon antenna array etalons. *ACS Photonics* **6**, 2917–2925 (2019).

[7] Munkhbat, B., Wróbel, P., Antosiewicz, T. J. & Shegai, T. O. Optical constants of several multilayer transition metal dichalcogenides measured by spectroscopic ellipsometry in the 300–1700 nm range: High index, anisotropy, and hyperbolicity. *ACS Photonics* **9**, 2398–2407 (2022).

[8] Baranov, D. G., Krasnok, A., Shegai, T., Alù, A. & Chong, Y. Coherent perfect absorbers: Linear control of light with light. *Nat. Rev. Mater.* **2**, 1–14 (2017).

[9] Zanotto, S. Weak coupling, strong coupling, critical coupling and fano resonances: A unifying vision. In *Fano Resonances in Optics and Microwaves*, 551–570 (Springer, 2018).

[10] Krasnok, A. *et al.* Anomalies in light scattering. *Adv. Opt. Photonics* **11**, 892–951 (2019).

[11] Thomas, P. A., Menghrajani, K. S. & Barnes, W. L. All-optical control of phase singularities using strong light-matter coupling. *Nat. Commun.* **13**, 1–6 (2022).

[12] Zanotto, S. *et al.* Perfect energy-feeding into strongly coupled systems and interferometric control of polariton absorption. *Nat. Phys.* **10**, 830–834 (2014).
